# Innovative landing zones for one-piece, rigidly fixated patient-specific subperiosteal implants in dental rehabilitation of severe maxillary and midfacial defects

**DOI:** 10.1186/s13005-025-00549-y

**Published:** 2025-12-18

**Authors:** Nils-Claudius Gellrich, Philippe Korn, Philipp Jehn, Fritjof Lentge, Michael-Tobias Neuhaus, Björn Rahlf

**Affiliations:** Department of Oral and Maxillofacial Surgery, Hannover Medical School, Carl- Neuberg-Str. 1, Hannover, 30625 Germany

**Keywords:** Pterygoid process, Dental implants, Patient-specific subperiosteal implant, Selective laser melting, CAD/CAM, Rigid fixation

## Abstract

**Purpose of the study:**

This study demonstrates and investigates the feasibility of a novel approach for dental rehabilitation in complex maxillary and midfacial defects, often resulting in severe pseudo-class III relationships. It explores the use of patient-specific subperiosteal implants (IPS Implants^®^ Preprosthetic) with innovative design variations that anchor to the skull base from below and laterally, extending beyond traditional implants to provide enhanced stability in cases of significant bone loss.

**Methods:**

Patients with significant maxillary and midfacial bone loss due to postablative defects were included in our case series, with or without irradiation. From 100 patients, 13 required design modifications based on FEM analysis, involving implant anchorage to the lateral skull base and/or pterygoid process. Eight patients received single-sided lateral skull base extensions; one posttraumatic case required bilateral extensions. In five of 13 cases, additional extensions to the pterygoid process were implemented. One case needed both bilateral skull base and pterygoid extensions, while two had bilateral pterygoid and a single-sided skull base extension. Outcomes assessed included primary stability, prosthodontic restoration, complications, and soft tissue management.

**Results:**

No implant failures occurred during follow-up, with functional stability established immediately post-insertion. Prosthodontic restoration succeeded in all cases, with no stability loss or periimplantitis. Mucositis was noted around posterior posts in some instances, and one bar required trimming due to soft tissue changes, without affecting implant function. One implant had to be removed, due to chronic pain.

**Conclusions:**

Patient-specific IPS Implants^®^ Preprosthetic with design extensions provide a biologically suitable solution for stable dental rehabilitation in severe maxillary and midfacial structure loss, without biomechanical limitations. Achieving rigid fixation distant from the soft tissue transition is crucial for success.

## Introduction

### General context

 All current protocols for dental rehabilitation in patients with significant loss of maxillary and midfacial structures due to tumor, trauma, or dental disorders rely on combined soft tissue and hard tissue (microvascular or non-vascularized bone) reconstruction before dental implant placement [1]; in more advanced approaches, even single-stage or two-stage ectopically inserted dental implants can be placed vector-wise guided into the fibula [2–8]. Actually, it was the group led by Dennis Rohner and Beat Hammer that initiated the concept of a comprehensive mandibular versus maxillary reconstruction procedure, which was initially entirely analogous in the beginning [9]. This protocol not only emphasizes the restoration of bone and soft tissue but also incorporates a comprehensive dental rehabilitation plan, integrating conventional dental implants into the workflow of planning and surgical rehabilitation, culminating in the fabrication of prosthodontic suprastructures that are mounted at time of microvascular tissue transfer from the fibula into the mandible versus the maxilla.

Depending on the chosen protocol, dental implants can achieve secondary stability either before the transfer of free tissue bone, as in the two-stage “Rohner protocol” or immediately after free microvascular bone transfer at the reconstruction site within the oral cavity in a one-stage protocol, such as “Jaw in a Day”. Both techniques allow for direct, although limited, loading when the suprastructure is mounted onto the dental implants. However, this reconstruction approach requires anchoring the dental implants near the original dentition prior to ablative surgery. Therefore, the placement of both bone and implants must be exceptionally precise to ensure alignment with the complex prosthodontic backward planning required.

### Problem

All these strategies still depend on adequate biological bone healing at the recipient site; without it, the entire dental rehabilitation protocol is at risk of failure [10]– [11].

Following maxillary ablation, there is often a functional or non-functional compensation of upper to lower lip interaction and lip competence, which may severely limit any dental rehabilitation protocol; additionally, radiation, immunotherapy and/or chemotherapy may present additional challenges to reconstruction [12]. In addition to addressing the maxillary or combined maxillary and midfacial defects, it is essential to consider the biomechanical relationship and interaction with the mandible, including its existing dentition or dental restorations [13]. Moreover, the presence and functionality of both intraoral and extraoral soft tissues, i.e. functioning subunits, play a crucial role in the overall success of the reconstruction [14]. This led to new design considerations, which have not been described before, in order to identify areas for safely anchoring the subperiosteal implant onto the skull base. Typically, both the outer midfacial frame - encompassing the root of the zygomatic arch and the lateral skull base - and the pterygoid process - even if partial resection has already occurred - were identified to allow for reliable extensions of the subperiosteal implant footplate, which can be safely placed transorally and rigidly fixated with 1.5 or 2.0 screws.

## Aim of the article

The authors developed a new and innovative type of patient-specific subperiosteal implants (IPS Implants^®^ Preprosthetic) based on the principle of functionally stable anchorage with immediate prosthetic initial treatment and non-restricted masticatory capacity [15, 16].

After starting the technique in 2014, the authors were confronted in 2020 with even more compromised anatomy due to maxillary ablation including extended necrosis from radiotherapy and lack of central midface vascularization. Therefore, design changes from already achieved master-designs in significantly atrophic maxillary cases had to be considered to anchor further away on adequately vascularized, well-enveloped bony tissues at the lateral and medial skull base.

Thus, the aim of this case series is to describe a new approach for maxillary dental rehabilitation and to characterize the included patients.

## Materials and methods

**Fig. 1:**
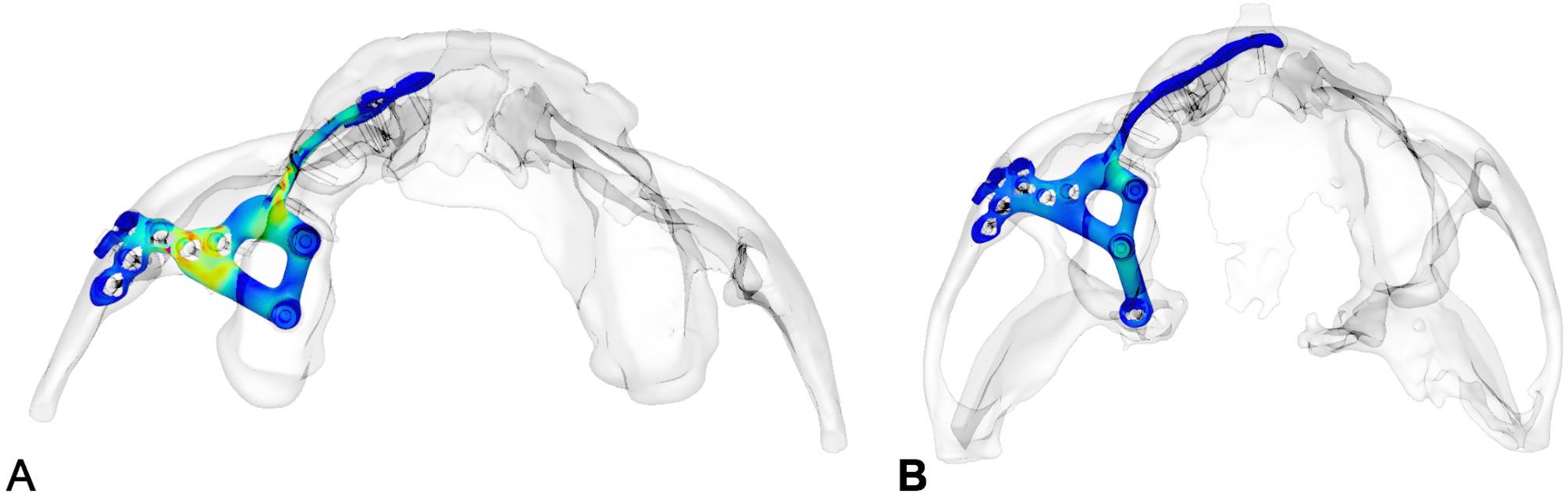
FEM-analysis of a preliminary IPS Implants^®^ Preprosthetic for reconstruction of right hemi-maxillary defect (**a**), with insufficient stability of the most posterior post. Revised design features an extension to the right pterygoid process as additional buttress (**b**), resulting in better load distribution in the FEM-analysis

Patients treated with IPS Implants^®^ Preprosthetic (KLS-Martin^®^, Tuttlingen, Germany) for dental rehabilitation between 2020 and 2024 in the Department of Oral and Maxillofacial Surgery of the Hannover Medical School have been screened for inclusion in this case study. Inclusion criteria were:

- Postablative defects of the maxilla or the midface.

- insufficient stability in preproduction FEM analysis (Fig. 1), using typical maxillary or midfacial buttresses for implant design

- modified IPS-p design with additional buttresses at the pterygiod process or the zygomatic arch

The study was conducted according to the declaration of Helsinki and was approved by the local ethics review committee (Nr. 8552_BO_K_2019). The clinics database and patients records were screened for patients treated with IPS-p. Those meeting the inclusion criteria were enrolled in this study.

The patients were routinely followed up and investigated in terms of:

- required soft tissue modifications prior to IPS Implants^®^ Preprosthetic (i.e. pre-, intra-, post-IPS Implants^®^ Preprosthetic placement)
- the number of screws used for rigid fixation in relation to the number of poles (ratio: screws per post)
- secondary interventions needed (e.g., soft tissue correction)
- infection
- fracture of the patient-specific implant
- prosthodontic suprastructure and masticatory function
- peri- and postoperative complications

Preoperative planning and implant design have been performed according to Gellrich et al.^17^ and Korn et al.^15^ using CBCT datasets and prosthodontic backwards planning.

The patients were followed up over a period of 9 to 52 months and investigated in terms of pre-IPS Implants^®^ Preprosthetic required soft tissue modifications (i.e. pre-, intra-, post-IPS Implants^®^ Preprosthetic placement), the number of screws used for rigid fixation in relation to the number of poles (ratio: number of posts/number of screws per case), and secondary interventions needed (e.g., soft tissue correction), infection, fracture of the patient-specific implant, prosthodontic suprastructure and masticatory function.

## Results

From a group of now 100 patients who were provided with an IPS Implants^®^ Preprosthetic for dental rehabilitation within the last 9 years, 13 patients were included in this case study (Table 1). These patients exhibited either extensive or configured defects in the maxilla and midface due to postablative defects (*n* = 7), with or without irradiation, to such an extent that stable anchorage at the typical maxillary and midfacial buttresses was not feasible, as determined by the FEM analysis. The follow up period ranged between 9 and 52 months with a medium follow up of 37,5 months.

**Table 1:** Distribution of cases includes diagnosis, number of patients, irradiation, use of microvascular and local soft tissue flaps, previous failures in bony reconstruction, prior dental implant treatments, and time since primary surgery

	indication	age at surgery	XRT	DIL	1	2	remaining dentition in operated jaw	restoration in the opposing jaw	follow up (month)
1	TU	55	x	--	--	RFF, LDF	A 12–16	A 36–47	62
2	TU	66	--	--	RFF* IC-f*	LDF	D	C 36–45	54
3	TU	46	--	--	RFF^#^	--	D	B 34, 32, 42, 44	45
4	TU (2x)	55	x (2x)	--	--	UAF, RFF	D	C 38–45	44
5	TU	65	x	--	RFF* LDF* FIB-f*	Abbé	D	B 34, 32, 42, 44	44
6	TU	57	x	--	LDF^#^	UAF	D	B 34 − 32	37
7	TU	65	x	--	LUFF^#^	--	D	A 36–47	33
8	cyst and oroantral fistula	34	--	--	--	LVP	C 14–24	A 36–47	18
9	atrophy (complex)	80	--	x	LBA-f*	--	A 15, 24, 26	A 36–46	37
10	atrophy (bisphosphonate)	69	--	--	--	--	D	A 36–45	16
11	ectodermal dysplasia	57	--	x	--	RFF	D	B 34, 32, 42, 44	33
12	trauma	68	--	x	4x IC-f*	RFF	D	B 36, 33, 46, 47	24
13	trauma	57	--	--	LUFF^#^ FIB-f IC-f	LDF LUFF	D	C 37–45, 47	41
average values		55							37,5

*DIL* Dental Implants Lost, 1 = Reconstruction related to the primary medical condition (* alio loco, # MHH), 2 = additional soft tissue transfer for separation of anatomical units prior to IPS Implants^®^ Preprosthetic (MHH), *LBA-f* local bone augmentation – failed, *IC-f* Ilia crest – failed, *FIB-f* Fibula – failed, *RFF* radial forearm flap, *LUFF* lateral upper arm free flap, *LDF* latissimus dorsi flap, *LVP* local vestibuloplasty

A: Prosthetically, fixed-supported denture, regions according to the FDI Tooth Numbering System

B: prosthetic, removable denture, abutment teeth or implants according to the FDI Teeth Numbering System

C: own teeth

D: no remaining natural teeth

In 2 cases, there were extensive posttraumatic defects of the upper jaw and midface: in one of these cases, there was a severe bilateral midfacial defect following a gunshot injury with complete loss of the upper jaw and central midface, including the nose and both orbital floors.

Four additional cases included an extensive cyst resulting in a large oroantral fistula, along with three massive atrophic maxillary cases exhibiting severe pseudo-class III due to ectodermal dysplasia, and two attributed to an incorrect primary conventional dental implant concept. In this latter case, the over-rigid rehabilitation of the mandible led to the failure of the upper implant-borne dental reconstruction and consequently, the underlying jaw bone.

The size and configuration of the defect necessitated a modification in design and expansion of the patient-specific subperiosteal implant for dental rehabilitation in all the aforementioned cases, with anchorage to either the lateral skull base, the pterygoid process, or both sides (Table 2). Maxillary defects have been categorized in partial maxillectomy, maxillectomy and atrophy defects (Table 2).

**Table 2:** Indications for modified implants, types of modifications (preauricular, pterygoid process), including screw and post number, screw-to-post ratio, and types of prosthodontic restorations

	indication	defect	pre-auricular extension (L = left; *R* = right)	pterygoid extension (L = left; *R* = right)	no. of screws	no. of posts	ratio screws per post	prosthodontic rehabilitation
1	TU	B 11–28	L	--	19	3	6,33	telescope
2	TU	A 18–28	R	--	21	4	5,25	bar
3	TU	B 11–18	R	--	24	3	8	bar
4	TU (2x)	A 18–28	R	R	18	4	4,5	bar
5	TU	A 18–28	R	R + L	21	4	5,25	bar
6	TU	B 11–18	R	R	18	4	4,5	bar
7	TU	A 18–28	R	R + L	19	4	4,75	temporary prosthesis srew ret.
8	cyst and oroantral fistula	B 15–18	--	R	12	2	6	screw
9	atrophy (complex)	C 18–28	R	--	21	4	5,25	bar
10	atrophy (bisphosphonate)	C 18–28	--	L	25	4	5,25	bar
11	ectodermal dysplasia	C 18–28	--	R + L	22	4	5,5	bar
12	trauma	A 18–28	--	R + L	25	4	6,25	bar
13	trauma	A 18–28	R + L	R + L	21	4	5,25	bar
average values					20,5	3,7	5,5	

Defects

A: Maxillectomy, regions according to the FDI Tooth Numbering System

B: Partial maxillectomy, regions according to the FDI Tooth Numbering System

C: Atrophy, regions according to the FDI Tooth Numbering System

Eight patients received a single-sided midfacial outer frame extension to the lateral skull base, with one posttraumatic case requiring bilateral extensions. Additionally, in five of the nine patients with outer midfacial frame extensions, supplementary extensions onto the pterygoid process were incorporated. Furthermore, one posttraumatic case necessitated bilateral lateral skull base extensions along with bilateral pterygoid process extensions, while two cases involved bilateral pterygoid extensions combined with a single-sided lateral skull base wing extension. Postoperative complications are summarized in Table 3, with postoperative pain being the most frequent. Two patients deceased during follow up period. In one case the implant had to be removed, due to chronic pain and infection.

**Table 3:** Presentation of the frequency of postoperative complaints or wound healing disturbances and complications during the follow-up period

	indication	postoperative pain	wound healing disorders	pain	infections	loss	Patient deceased
1	TU	x	--	--	--	--	--
2	TU	--	--	--	--	--	--
3	TU	x	--	--	--	--	--
4	TU (2x)	--	--	--	--	--	--
5	TU	--	--	--	--	--	x
6	TU	--	--	--	--	--	--
7	TU	x	--	--	--	--	--
8	cyst and oroantral fistula	x	--	x	x	x	--
9	atrophy (complex)	--	--	--	--	--	--
10	atrophy (bisphosphonate)	x	--	--	--	--	--
11	ectodermal dysplasia	--	--	--	--	--	--
12	trauma	x	--	--	--	--	--
13	trauma	--	x	--	--	--	x
overall numbers		6	1	1	1	1	2

Three patients had already undergone an attempted extensive bone transplantation, which had failed. Similarly, conventional dental implants had been placed for dental rehabilitation in three patients, but had to be subsequently removed, or a long-term successful therapy could not be achieved thereafter.

In Tables 1 and 2, the 13 patients are listed who have so far received design modifications to the skull base due to situations that could not be otherwise biomechanically addressed by an IPS Implants^®^ Preprosthetic, as determined by the FEM (Finite Element Method) analysis, for functional dental immediate rehabilitation.

## Clinical cases

### Case no 1

**Fig. 2:**
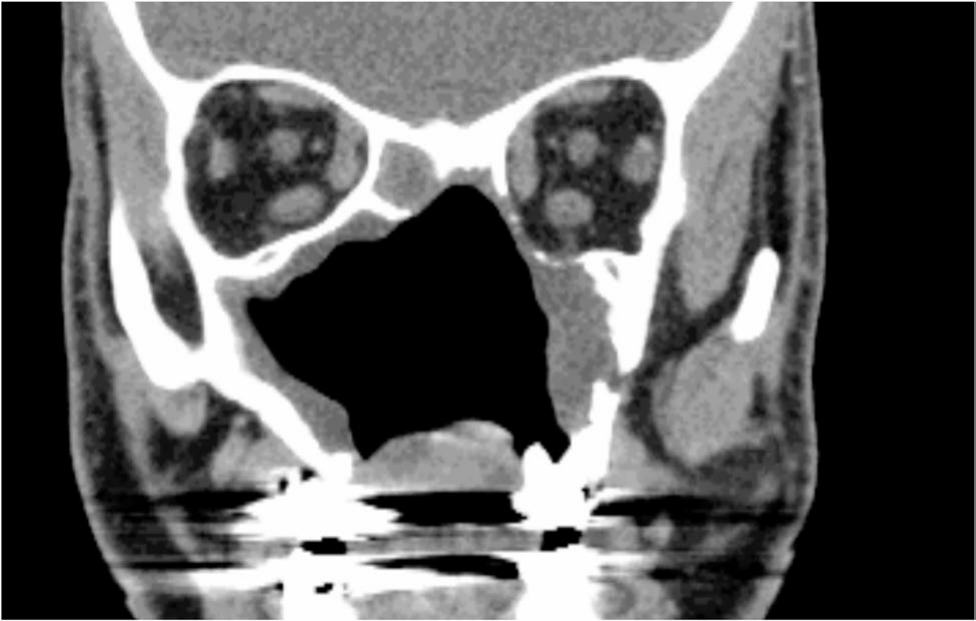
Following radical ablation and adjuvant curative intended radiotherapy elsewhere, in addition to the left lateral maxillary sequestration, the coronal view of the CT scan reveals an exenterated central midface defect with sclerotic bone formation and partial loss of the left orbital walls, along with a rounded inferior rectus muscle

**Fig. 3:**
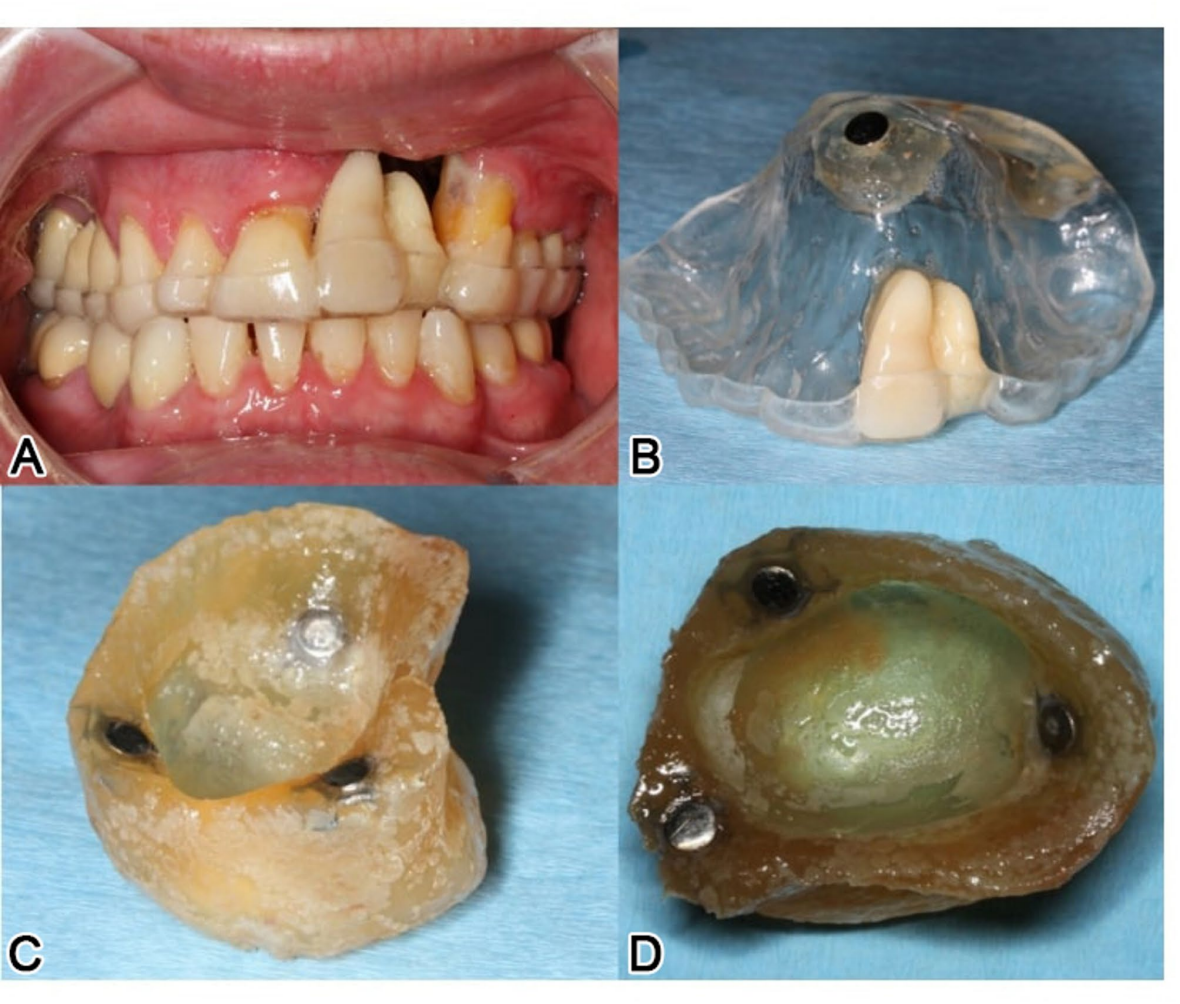
Intraoral view (**a**) of the patient in Fig. 1 with the individual attempt of a temporary combined obturating and multipart dental prosthesis (**a**-**d**) (note: magnetic elements helped stabilize the multi-piece construction)

A 46-year-old male patient, post-central midface ablation and adjuvant radiotherapy, presented with osteoradionecrosis due to poor revascularization from extensive bilateral midfacial resection. He used a three-part obturating device with magnetic retention between sections (Figs. 2 and 3). The referral sought a solution for dental rehabilitation without an obturator.

**Fig. 4:**
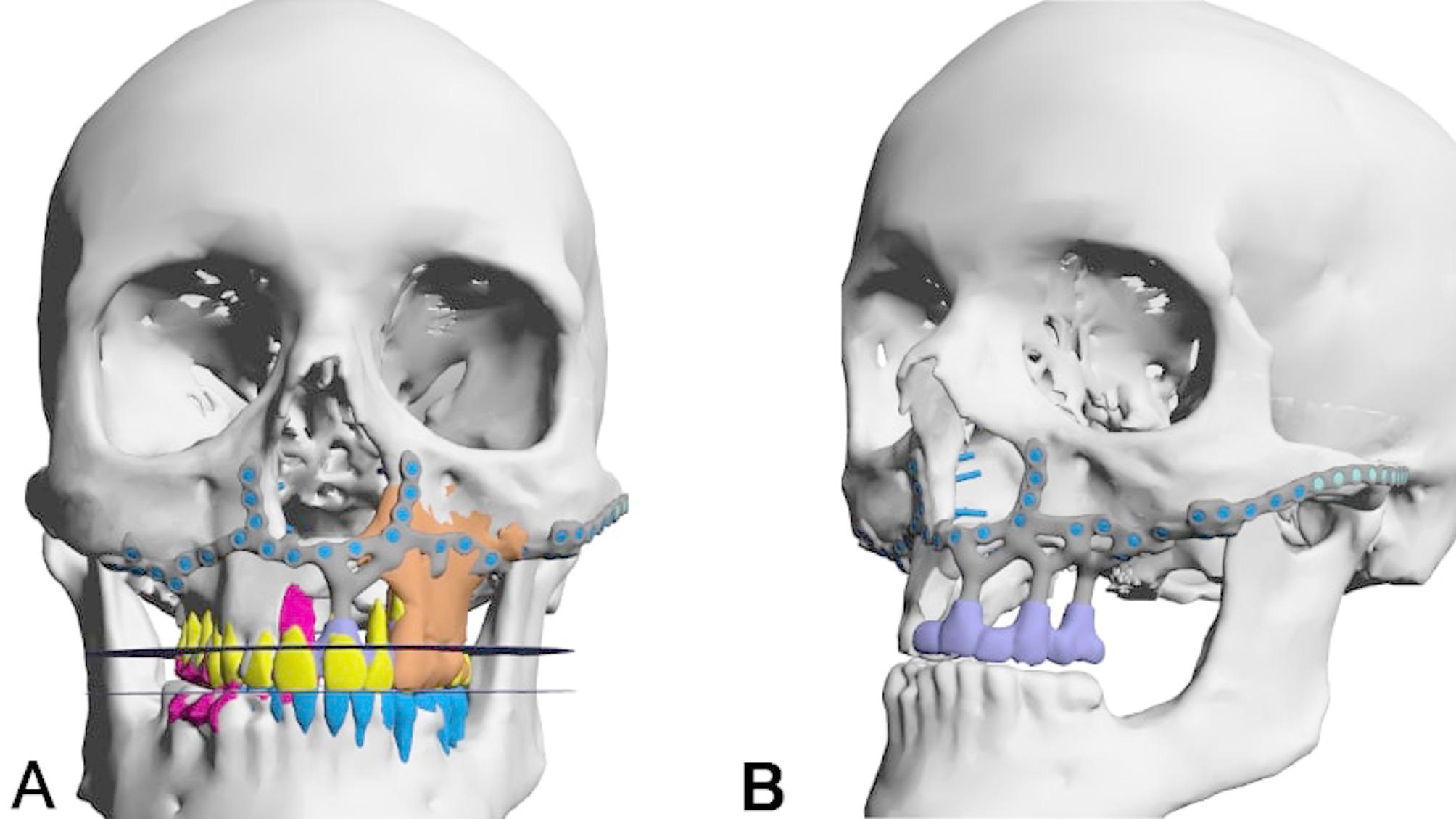
**a - b**: Two views from the interactive PDF case planning (patient from Figs. 1 and 2) of the IPS-Gate (KLS Martin Group, Tuttlingen, Germany). In the enface (**a**) and oblique lateral view (**b**); the virtually dentate patient is depicted in (**a**) where the segmented left maxilla (orange) shows the area of unclear vascularity; the first provisional prosthesis, designed with a high-water level, is shown mounted onto the IPS Implants^®^ Preprosthetic in (**b**). Notice: not any hole displayed is expected to be mounted with a screw

**Fig. 5:**
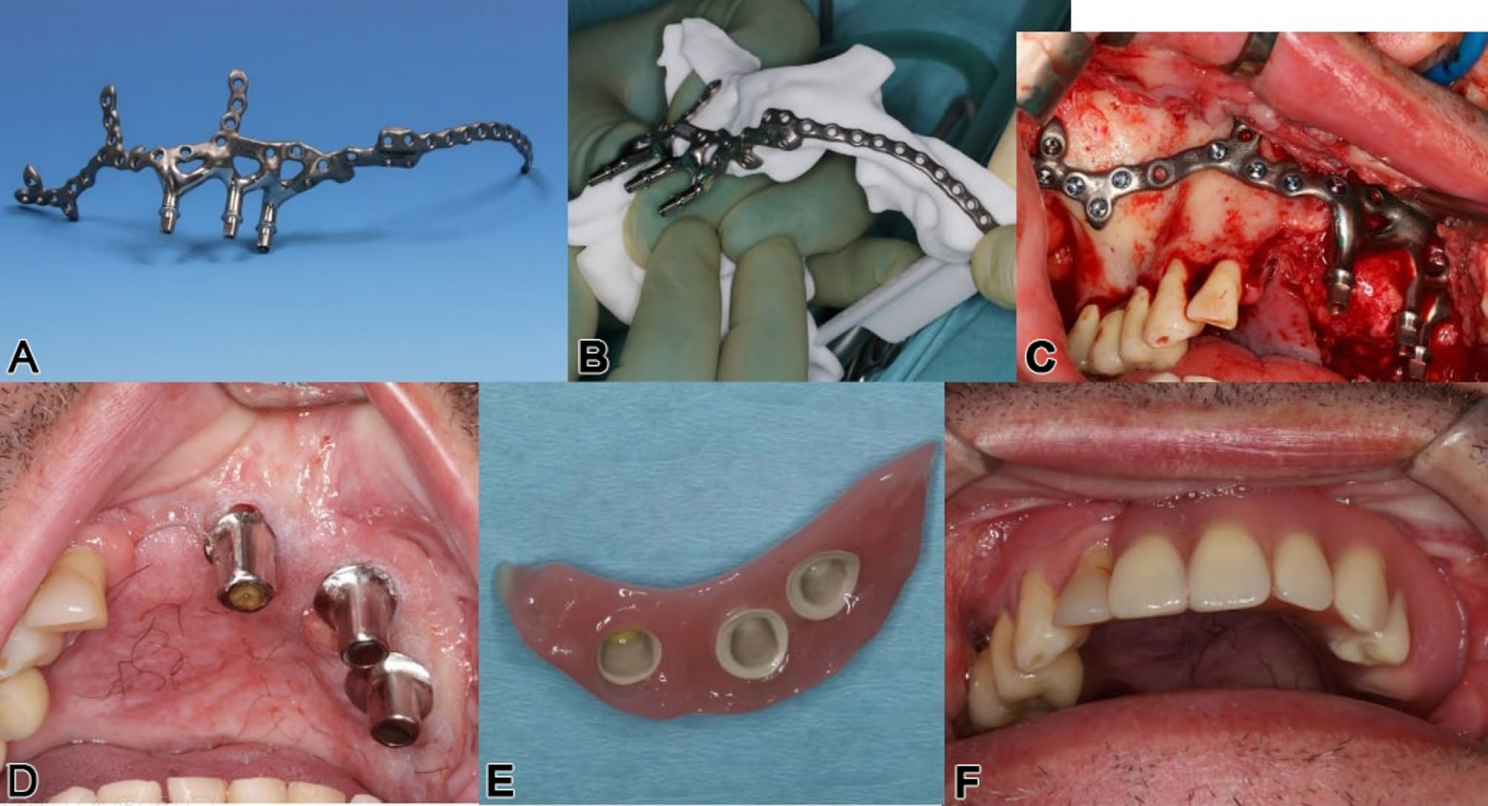
**a – f**: Preoperatively, the isolated IPS Implants^®^ Preprosthetic from the patient in Fig. 3 with three posts is displayed in (**a**), and mounted intraoperatively onto the autoclaved patient’s biomodel showing the long extension to the left lateral skull base (**b**). The insertion and screw fixation are provided transorally versus preauricularly at the root of the zygomatic arch (**b**, **c**); telescopes are used as primary crowns (**d**) for the final suprastructure, which is shown in (**e**) & (**f**). [note: the additionally placed radial forearm flap allowed for the rehabilitation of the vestibular side to the lip, whereas the latissimus dorsi flap served to obliterate the devascularized central midface and to reconstruct the horizontal subunit of the hard palate (**d**)]

Clinical and volume analysis showed osteonecrosis and bone sequestration in the left maxillary area, limiting safe anchorage due to poor vascularization (Fig. 2). A two-stage surgical approach was planned: first, a latissimus dorsi myocutaneous flap was used to fill the midfacial cavity after necrotic tissue removal, creating a horizontal palatal subunit and separating the oral and nasal cavities. A second microvascular radial forearm flap restored the lip and vestibule, allowing safe placement of a subperiosteal implant between the flaps without disrupting vascularization (Figs. 4 and 5d). To anchor the implant securely, an extension to the lateral skull base was designed for ipsilateral fixation (Figs. 4a and b and 5a and b).

A digitally designed titanium implant, manufactured with selective laser melting, was transorally placed under general anesthesia. The approach included subperiosteal dissection to the malar prominence and preauricular dissection at the skull base (Fig. 5c). Real-time navigation guided drilling was used to ensure accurate screw insertion. Three months post-surgery, the high-water provisional structure seated on three posts was upgraded to a telescoping modular cover denture, combining overdenture benefits with optimal hygiene (Fig. 5e, f). The subperiosteal implant allowed immediate loading, with telescoping abutments and crowns supporting a hard-palate-free modular coverdenture (Fig. 5d, e).

The final prosthesis was planned for three to six months post-implant, allowing for soft tissue stabilization. This patient required no bone grafting but significant soft tissue reconstruction to revascularize and restore midfacial subunits for effective implant placement.

## Case no 2

**Fig. 6:**
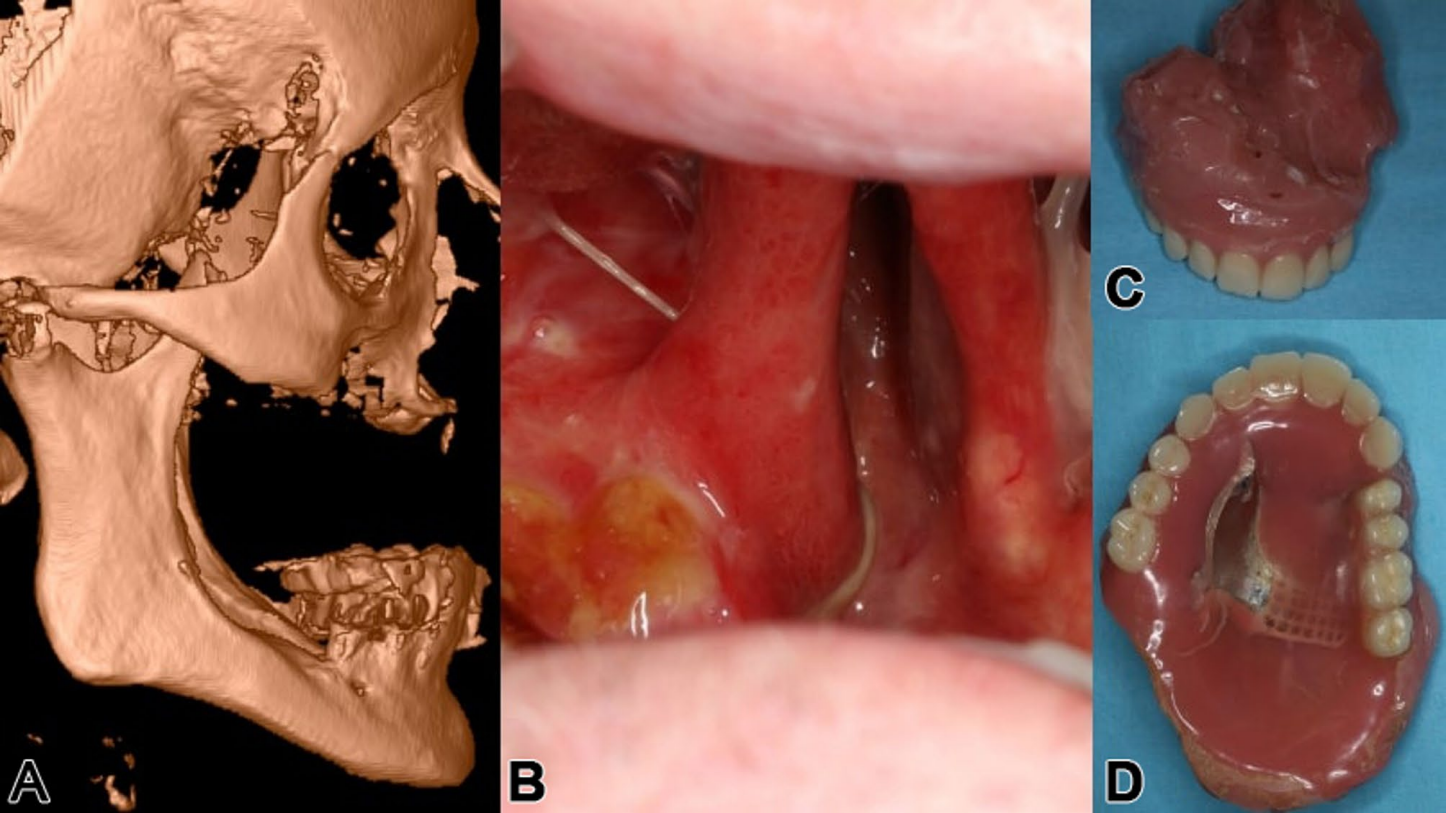
**a – d**: Preoperatively, a lateral 3D view from the right side of the helical CT scan is displayed in (**a**); intraorally, the extensive defect with the absent hard (and soft) palate is visible (**b**); the large obturating maxillary defect prosthesis is shown in (**c**) & (**d**) (note: this prosthesis was non-functioning in the affected patient)

**Fig. 7:**
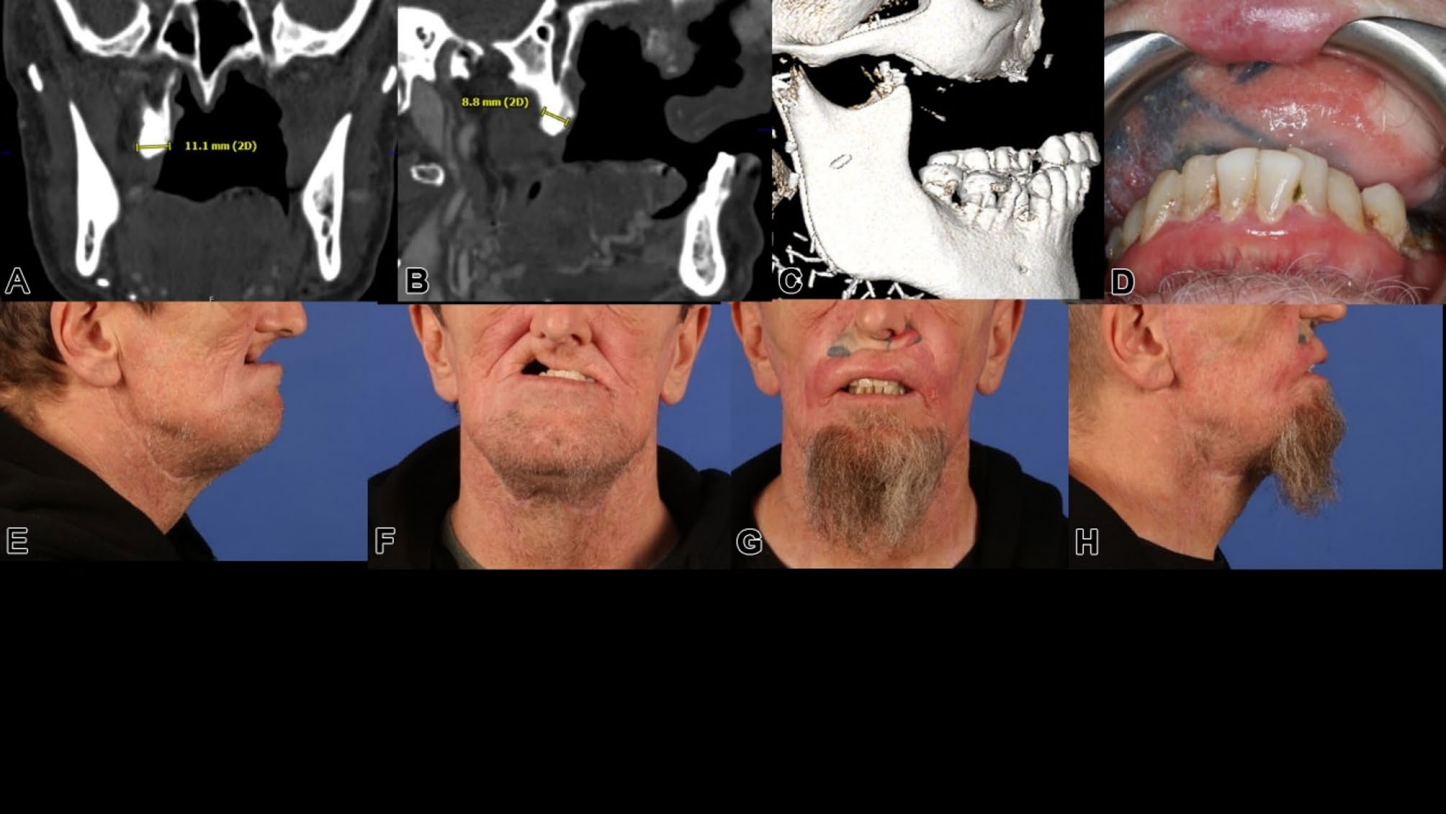
**a** – **h**: Preoperatively, before (**a**, **b**, **e**, f) and after microvascular soft tissue reconstruction (**c**, **d**, **g**, **h**) of ablated intraoral horizontal and intra-/extraoral vertical anatomical subunits. Two plane views (coronal (**a**) and sagittal (**b**)) display possible bony anchorage on the right pterygoid process; the left pterygoid process is completely missing. The extended pseudo-class III is strikingly obvious (**b**, **e**, **f**), especially with the radiopaque template for the intended maxillary dental rehabilitation (**c**); after lateral upper arm free flap (for horizontal subunit of hard and soft palate) and split radial forearm free flap (for intra- and extraoral vertical subunit of lip and vestibule) reconstruction (**d**, **g**, **h**), the preexisting postablative huge pseudo-class III is nearly compensated

A 55-year-old male, treated within 17 years for adenoid cystic carcinoma and secondary squamous cell carcinoma with two radical resections and each 65 Gy of radiotherapy, presented with unreconstructed soft and hard tissues, leaving him without a functioning palate, unable to eat or speak effectively. His upper lip was significantly retracted, with a deformed nose due to missing nasal support (Figs. 6 and 7a-f). The DICOM analysis with a radiopaque template revealed a large vertical discrepancy between the existing and intended anatomy (Fig. 7c), and no stable points were available for osteosynthesis (Figs. 6a and 7a-c).

**Fig. 8:**
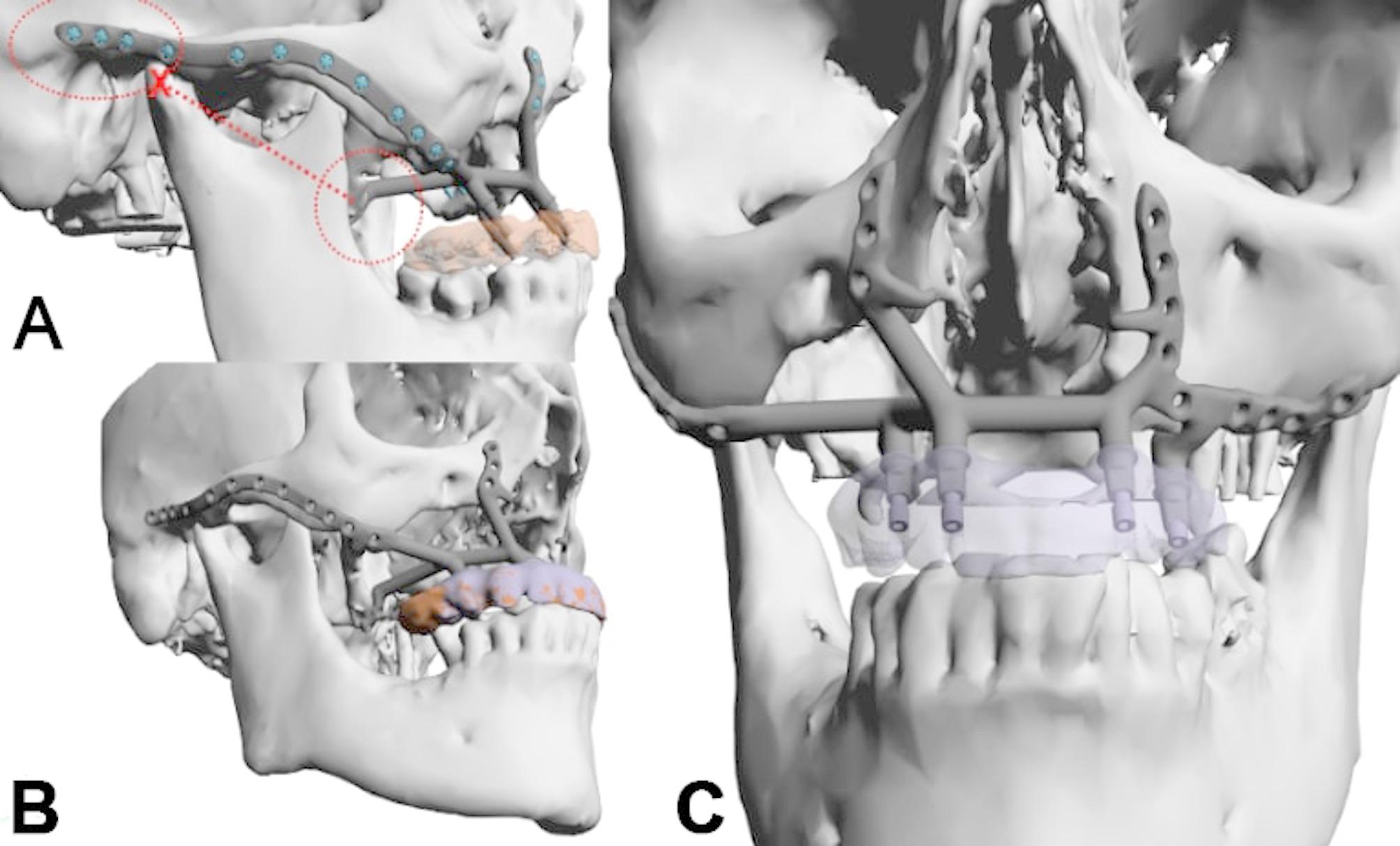
**a – c**: Three views from the interactive PDF of the IPS-Gate (KLS Martin Group, Tuttlingen, Germany). In the lateral view (**a**, **b**), the postablative condition is visible with a massive pseudo-class III and intended provisional dental prosthesis (**a-c**); the IPS Implants^®^ Preprosthetic is in place, with dotted encirclements showing the anchorage on the right pterygoid process and the lateral skull base (a red cross marks the condylar head) (**a**). The en face view (**c**) and lateral view (**b**) demonstrate additional 3D features of the patient-specific subperiosteal implant, including small hooks around the piriform aperture (please note: a one-piece design is strongly recommended for single-jaw dental rehabilitation, and this is ensured with the subnasal connecting bar)

**Fig. 9:**
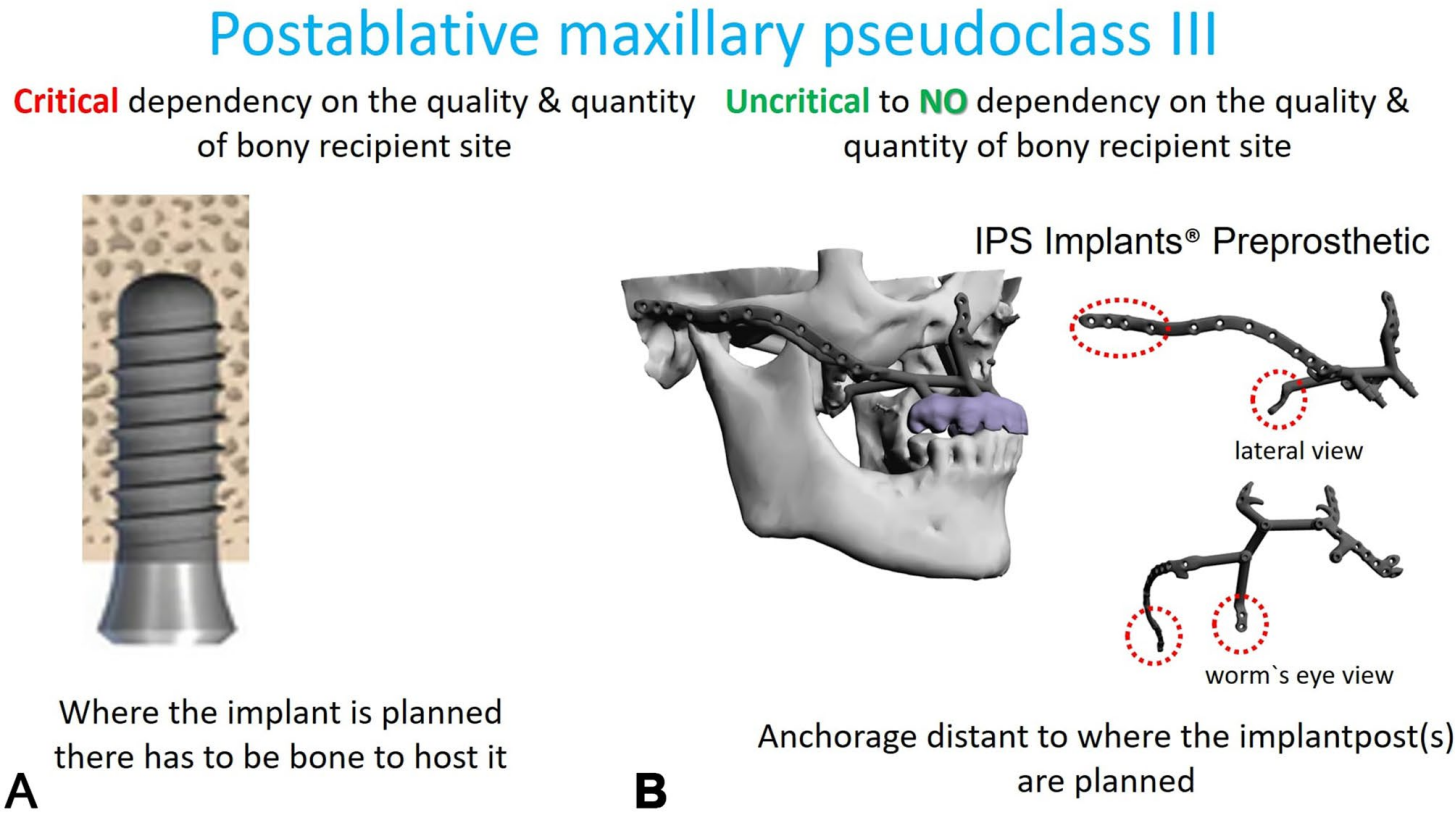
**a**, **b**: The single IPS Implants® Preprosthetic is displayed in (**a**) & (**b**), where the crucial design modifications are encircled with a red dotted line; these key modifications allow for more distant levering and functionally stable screw retention to enable a rigidly fixated subperiosteal implant that can be fully loaded primarily

**Fig. 10:**
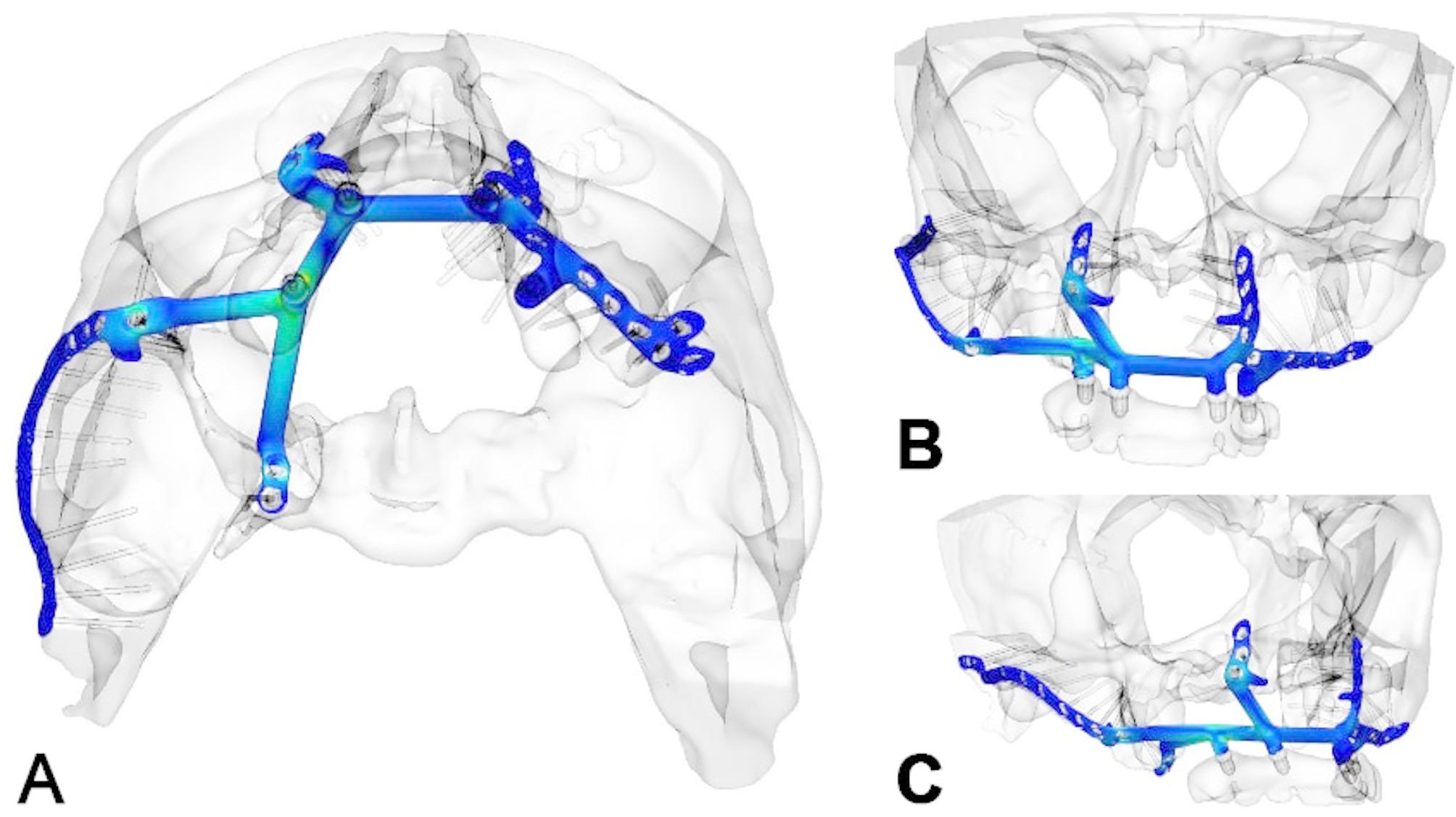
**a – c**: Three views of the heatmap from an FEM analysis of the implant planning (**a-c**); this ensures that the design is unlikely to result in biomechanical failure, and that four posts in combination with the displayed implant design are fully adequate to withstand the chewing forces and provide dental rehabilitation for the maxillectomy patient. The distant anchorage enables the utilization of better vascularized and more well-enclosed soft tissue lever zones

Initial surgery used two vascularized flaps—a radial forearm and a lateral upper arm free flap—to reconstruct the soft tissue, separate the oral from nasal cavities, and support the upper lip (Fig. 7g-h). Planning for IPS Implants^®^ Preprosthetic fixation identified secure points on the right zygomatic arch and pterygoid process (Figs. 8 and a and 9). FEM analysis ensured the implant met biomechanical needs (Fig. 10), with left-side anchorage points and extended right-side anchorage to reliable bone.

**Fig. 11:**
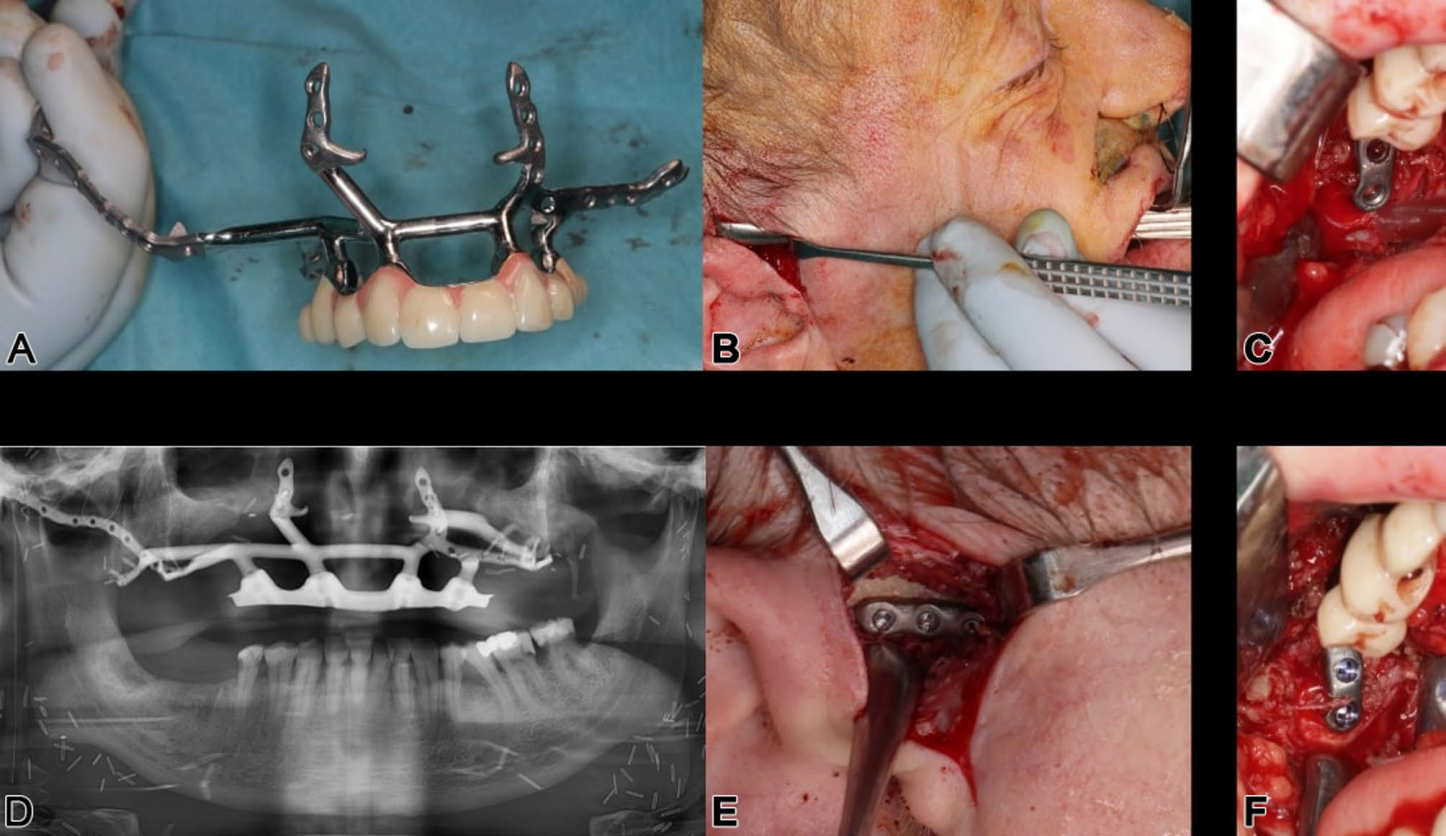
**a** – **f**: Intraoperative view of the IPS Implants® Preprosthetic and the mounted high-water provisional prosthesis (**a**); dissection transorally runs subperiosteally over the zygomatic arch to the ipsilateral preauricular release incision (**b**); the one-fit-only design can be controlled transorally on the pterygoid process (**c**) and lateral skull base (**e**); two screws provide rigid fixation on the right pterygoid process (**f**); the postoperative situation is displayed with the orthopantomogram (**d**)

**Fig. 12:**
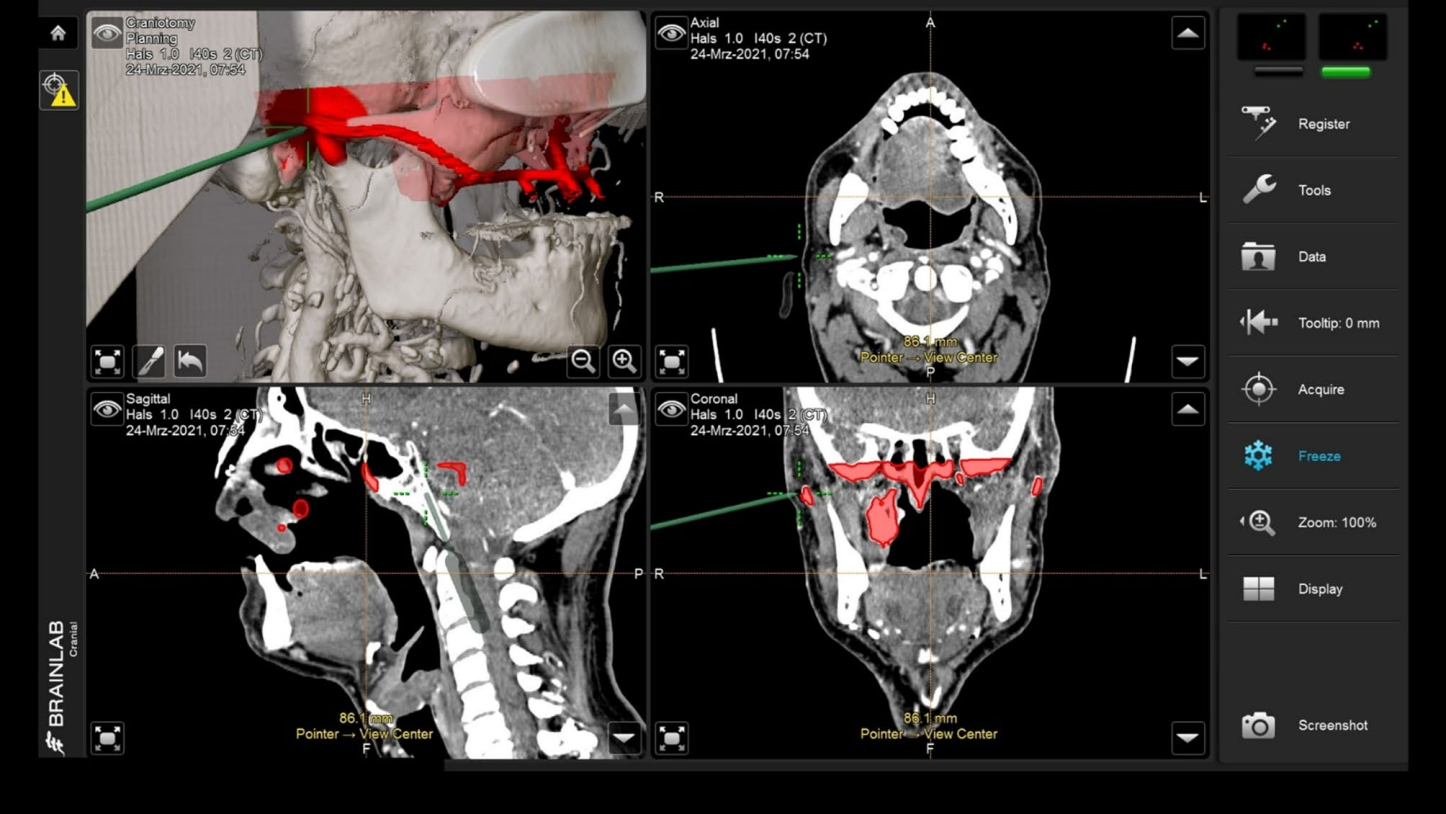
Screenshot of intraoperative navigation using the Curve^®^ System (Brainlab, Munich, Germany) during insertion of IPS Implants^®^ Preprosthetic with the pointer tracked at the root of the right zygomatic arch to validate real-time the available bone stock prior to drilling for screw retention. The STL model of the IPS Implants^®^ Preprosthetic is displayed in the four-field view in all three multiplanar perspectives (i.e., axial, coronal, sagittal) and a 3D view

Following soft tissue reconstruction, a subperiosteal implant with four posts and a right lateral wing was fixed to the skull base (Fig. 11). Real-time navigation assisted screw placement into the pterygoid process and skull base (Fig. 12). The implant’s connecting bar was housed in a vascularized soft tissue envelope to protect the titanium connector (Fig. 11a, d), allowing for easy cleaning and durable load-bearing.

**Fig. 13:**
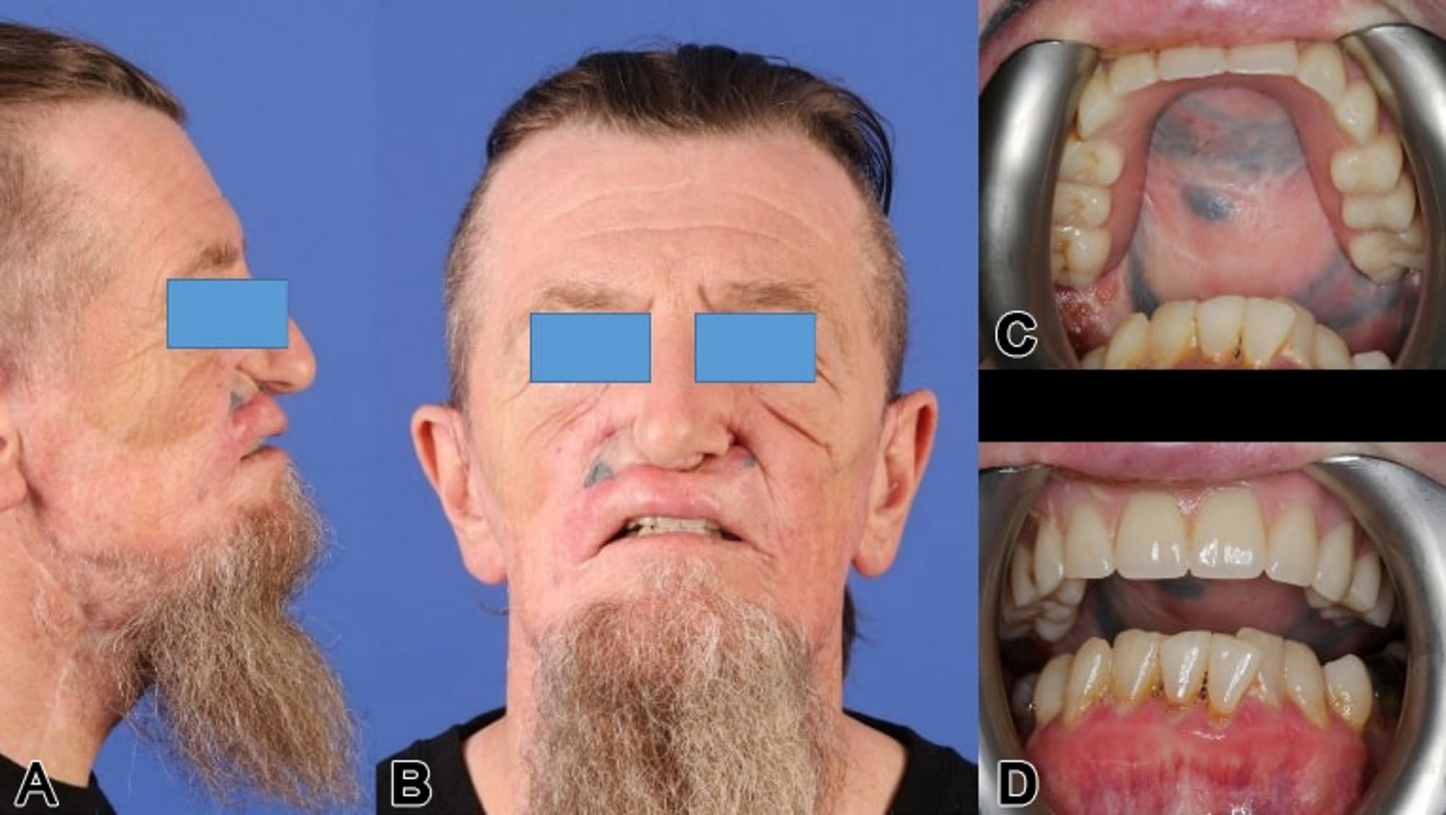
**a - d**: Two clinical extraoral views (**a**, **b**) show the patient definitively provided with dental prosthesis after completion of the surgical rehabilitation with microvascular reconstruction of vertical and horizontal anatomical subunits and integration of the IPS Implants^®^ Preprosthetic, with evidence of acceptable though not normal lip competency. The severe pseudo-class III malocclusion is compensated for without the need for bone grafting in the resected upper jaw and midface area. Two intraoral views (**c**, **d**) show the final bar-retained removable overdenture; the noticeable pigmentations subnasally and in the reconstructed palate result from the tattooing of the transferred microvascular autogenous flap grafts

The final maxillary dental rehabilitation used a bar-retained, hard-palate-free coverdenture extending from the first molar to first molar (Fig. 13). The patient is now fully functional with unrestricted eating and effective lip speech. This case highlights an innovative approach to addressing maxillary reconstruction in irradiated patients using soft tissue reconstruction and a subperiosteal implant to support a modular overdenture.

## Case no 3

**Fig. 14:**
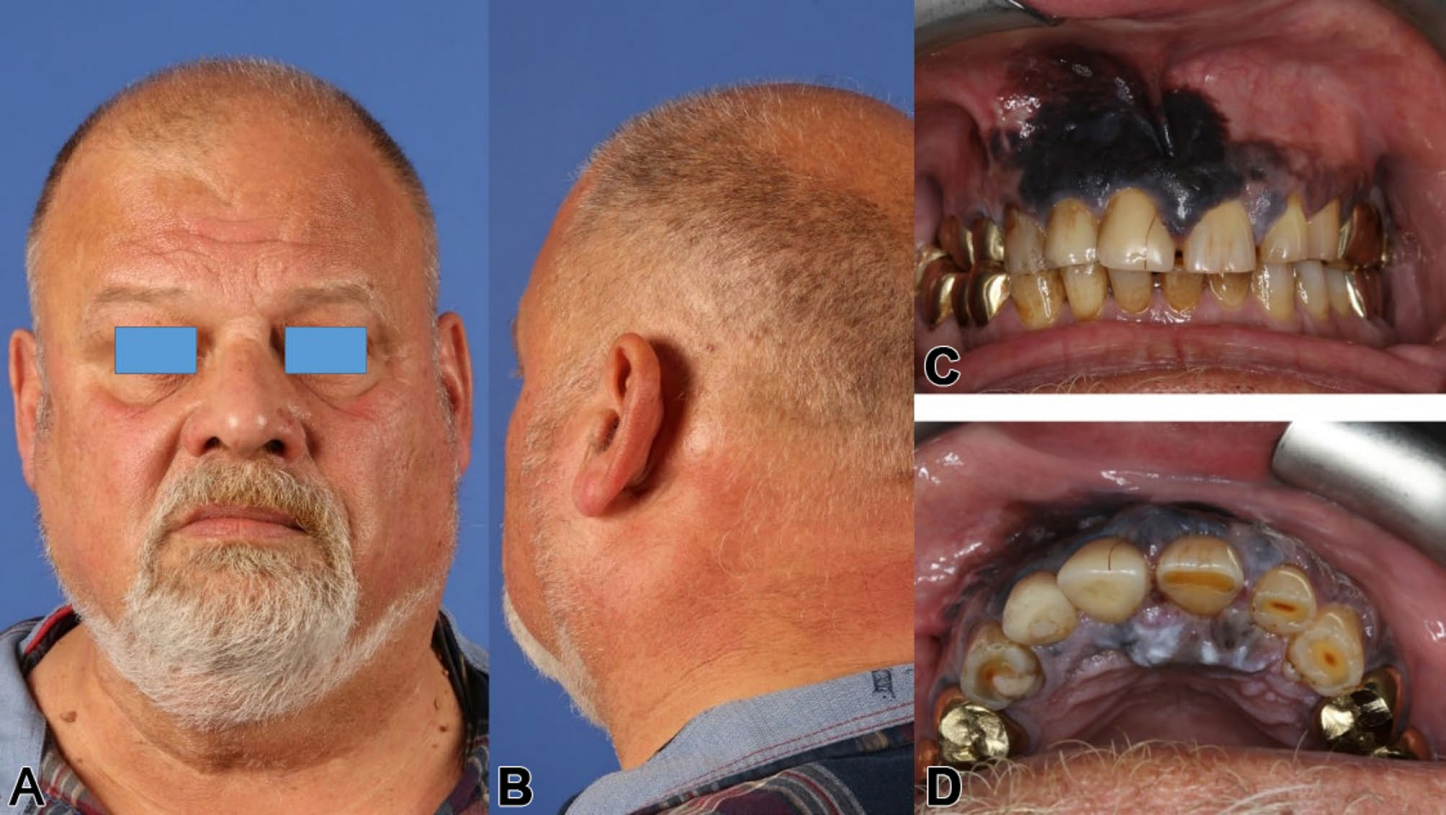
**a – d**: Preoperatively, a posterior lateral view from the left side of the patient with the pronounced left cervical metastasis (**a**) is depicted; intraorally, in the closed bite (**b**), and in the bottom view, the extensive malignant melanoma becomes apparent

**Fig. 15:**
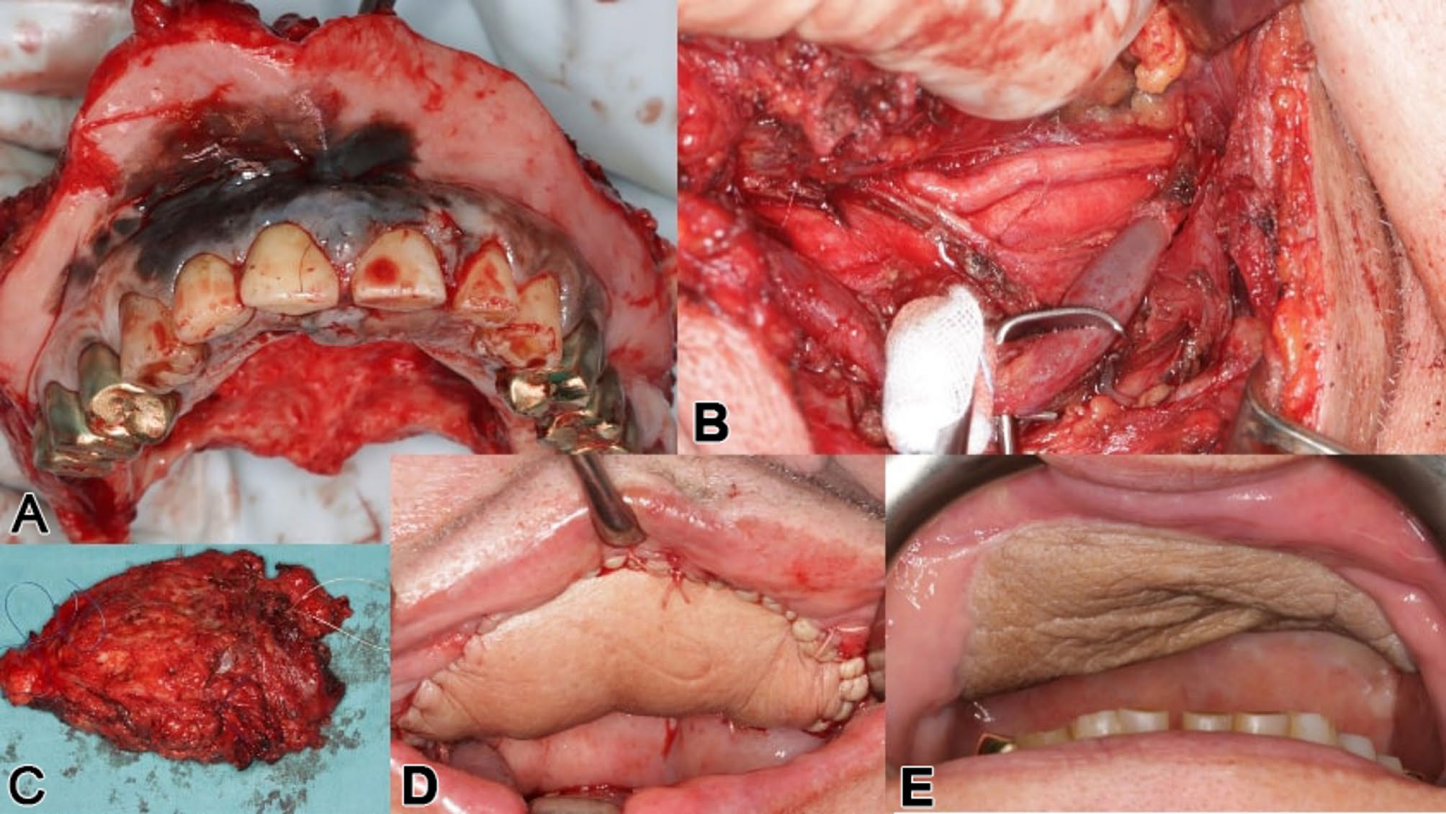
**a – e**: Intraoperatively, the tumor resection including the intended safety margin is visible as a complete maxillectomy (**a**); on the left cervical side, the internal jugular vein is dissected out, and the metastasis is removed as part of the left neck dissection (**b**, **c**); in (**d**), the microvascular lateral upper arm free flap is depicted as part of a delayed primary reconstruction, i.e., two-stage after obtaining definitive histology, and after integration (**e**)

A 65-year-old male with extensive maxillary melanoma underwent a full maxillectomy, bilateral neck dissection, and received radiotherapy, chemotherapy, and immunotherapy (Figs. 14 and 15). After resection, a thermoplastic template temporarily covered the defect, serving as a base for periodontal dressing. Reconstruction followed with a lateral upper arm free flap to create a vascularized barrier between the oral and nasal cavities, supporting the upper lip.

**Fig. 16:**
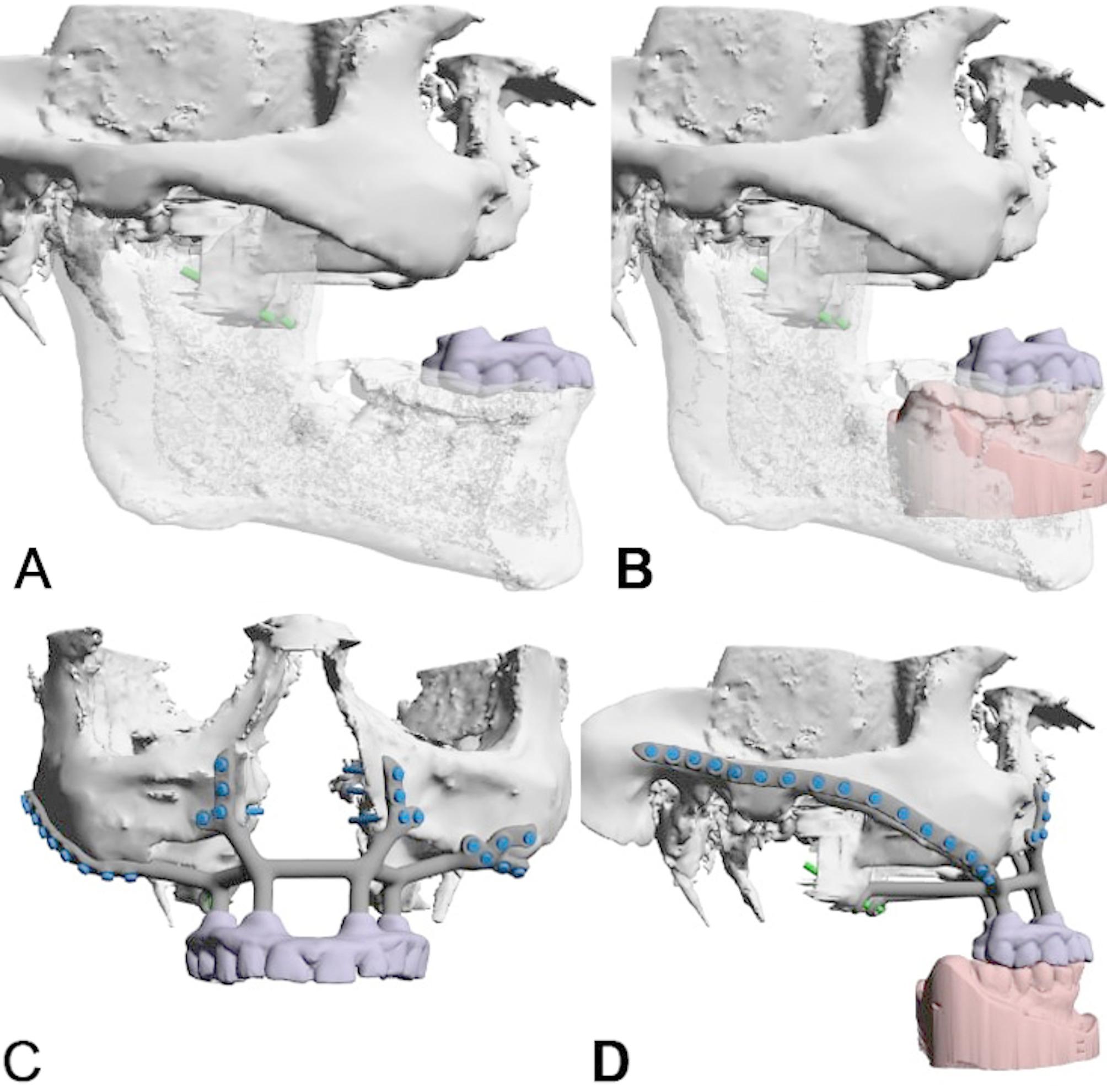
**a – d**: Four views from the interactive PDF from the IPS-Gate (KLS Martin Group, Tuttlingen, Germany). In the lateral view (**a**, **b**), the post-ablative condition is visible with massive pseudo-class III and intended provisional dental prosthesis (**a**) (please note: two green vectors represent the possible screw anchors in the bilateral pterygoid process area), accordingly, in (**b**), the scanned mandible model is matched to the cone-beam CT dataset. The additionally introduced IPS Implants^®^ Preprosthetic with the possible screw positions are shown in the frontal (**c**) and lateral views (**d**) (Note: The screw positions indicated above the zygomatic prominence and zygomatic arch are typically not utilized)

**Fig. 17:**
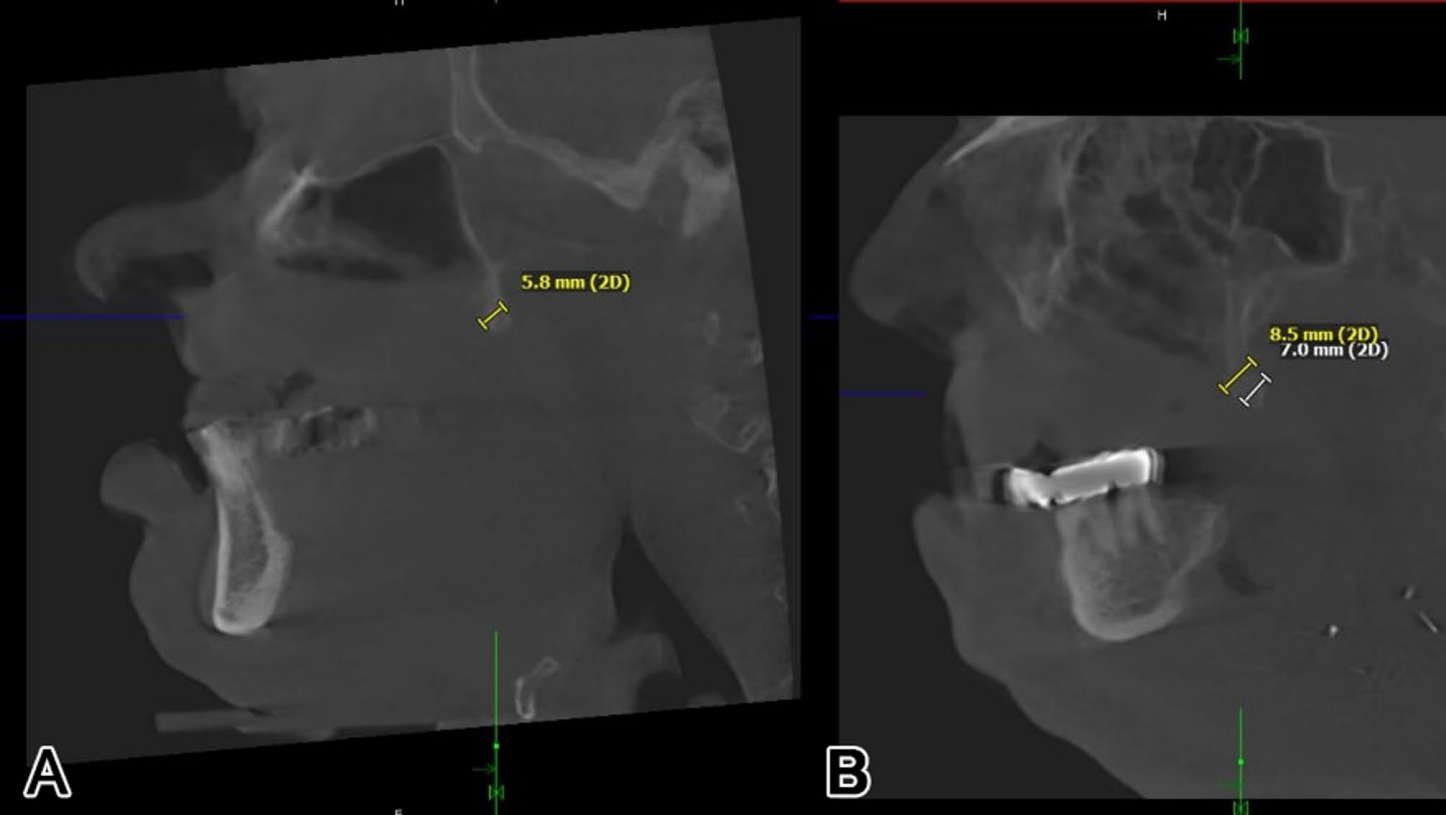
**a –b**: Two sagittal views of the cone-beam CT dataset. Potential anchorage for the left (**a**) and right pterygoid processes (**b**) is displayed

**Fig. 18:**
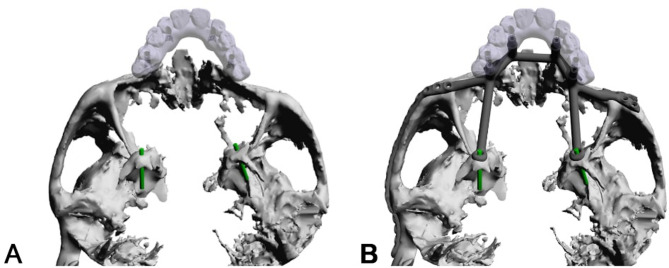
**a – b**: Two views of the virtual plan of the IPS Implants^®^ Preprosthetic. Following extended maxillary ablation, only the pterygoid processes bilaterally allow for safe bony anchorage (**a**). The framework implant is shown with the two long extensions to the pterygoid processes and the long bar extending to the right lateral skull base along the zygomatic arch (**b**)

**Fig. 19:**
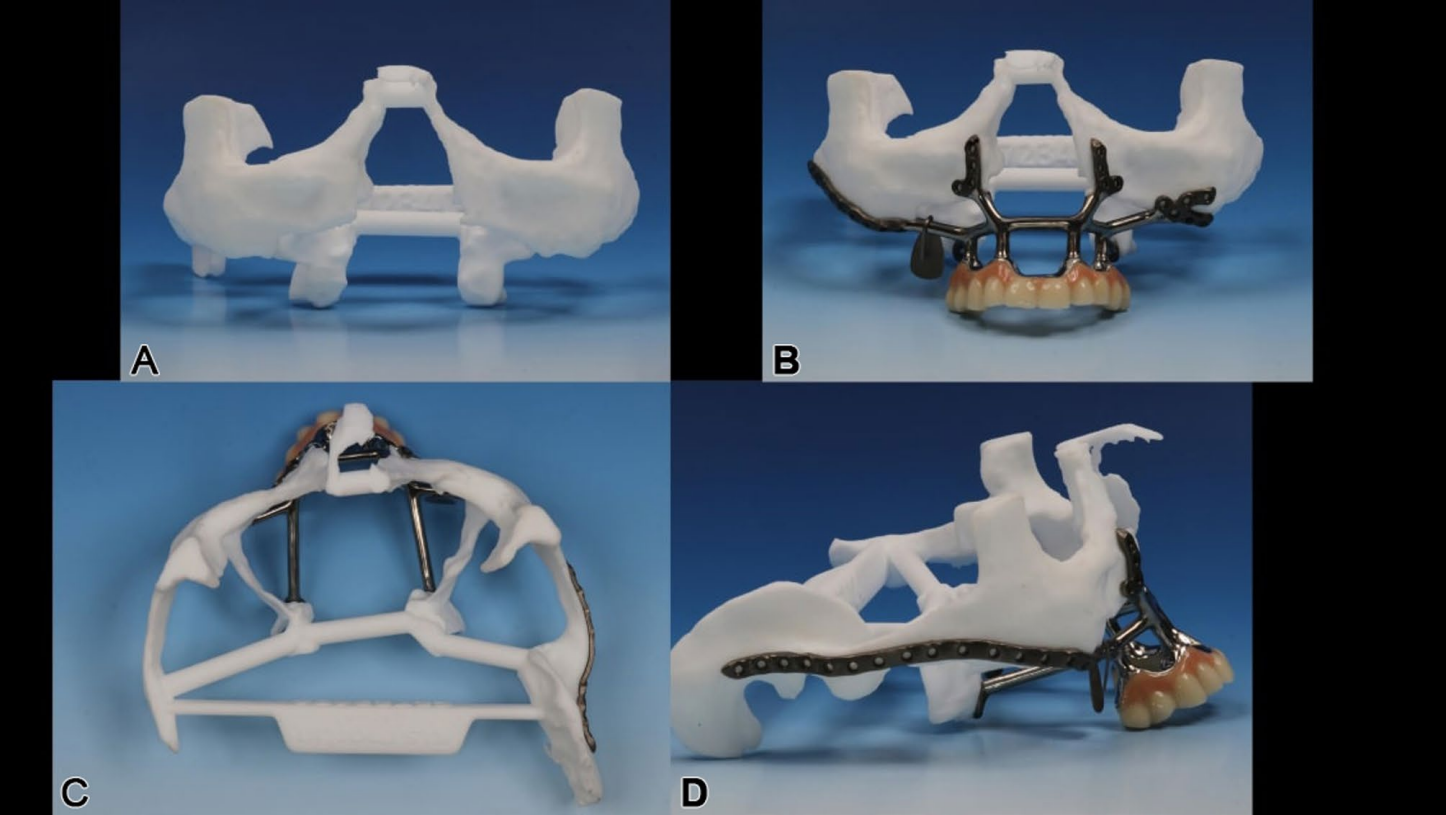
**a – d**: Two views of the IPS Implants^®^ Preprosthetic mounted onto the patient-specific biomodel from above (**a**) and from the right lateral view (**b**). (Important to note: the two extensions for anchoring on the two pterygoid processes as well as the long arm extending to the right lateral skull base)

**Fig. 20:**
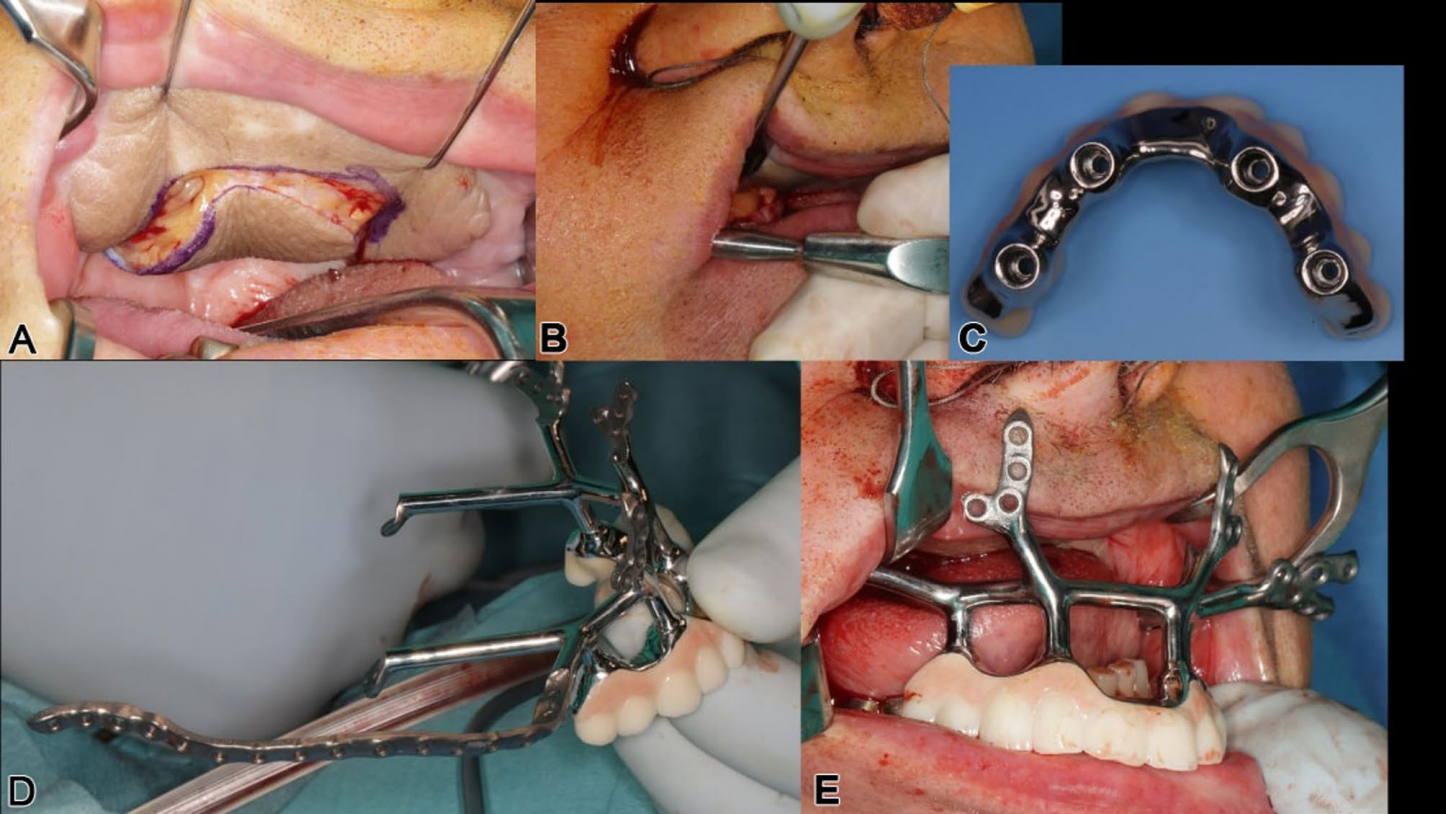
free **a** – **e**: Intraoperative view with incision made within the previously transplanted lateral upper arm free flap (**a**), transoral subperiosteal dissection along the lateral midface and right zygomatic arch to preauricular region (**b**); the provisional non-precious metal bar-supported dental prosthesis in high-water design was photographed intraoperatively from below (**c**) and screwed onto the IPS Implants® Preprosthetic intraoperatively (**d**) for better transoral insertion (**e**). (Please note: a separate preauricular extraoral approach of approximately 4 cm is required for drilling and screwing to enable functionally stable osteosynthesis in the lateral skull base area)

**Fig. 21:**
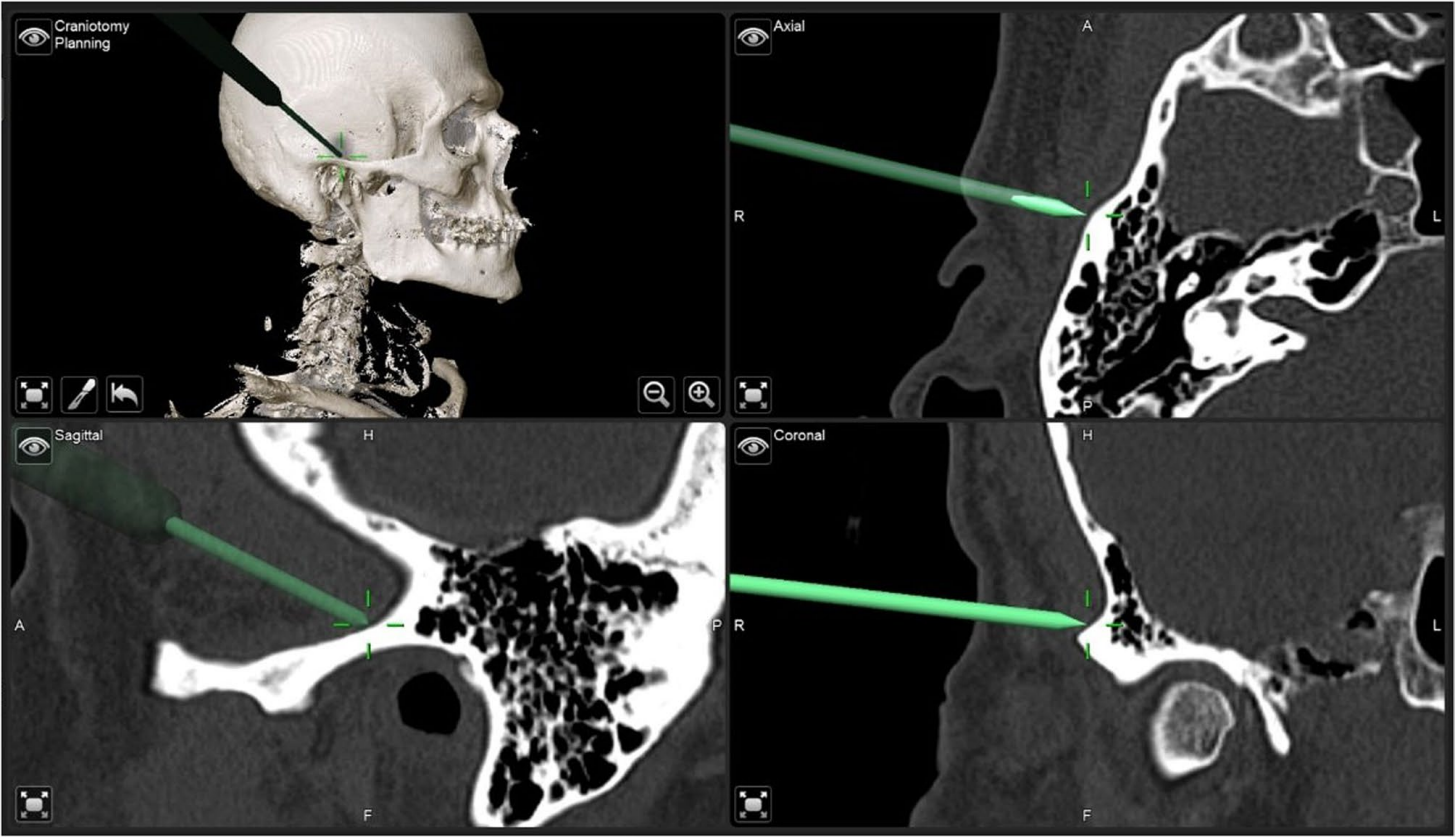
Screenshot of intraoperative navigation using the Curve^®^ System (Brainlab, Munich, Germany) at the lateral skull base to validate real-time the available bone stock prior to drilling for screw retention. Screenshot during navigation, displaying the four-field view in three multiplanar perspectives (i.e., axial, coronal, sagittal) and a 3D view

Nine months later, an IPS Implants^®^ Preprosthetic was digitally planned with extensions to the pterygoid processes and skull base for secure fixation (Figs. 16, 17, 18, 19 and 20). Preoperative imaging and prosthodontic backward planning helped determine implant position (Fig. 20). During surgery, the one-piece implant was inserted under general anesthesia, though scarring from prior treatments posed challenges. Intraoperative navigation was used to safely position screws in the pterygoid processes and skull base (Fig. 21).

**Fig. 22:**
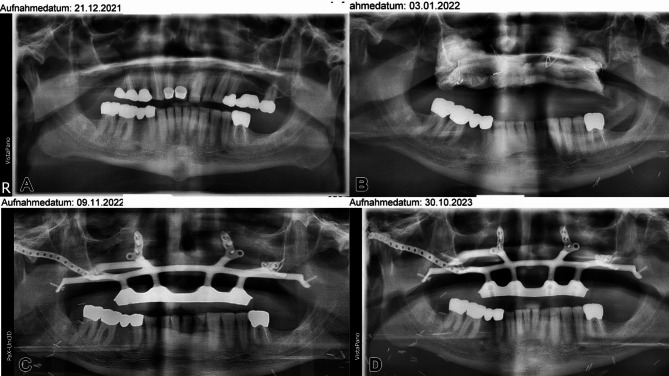
**a – d**: Four orthopantomograms show the progression of the patient with a metastasized malignant melanoma of the entire maxilla before resection (**a**), after resection and before microvascular soft tissue reconstruction of the functional subunits postablation using a lateral upper arm free flap (**b**), after insertion of the IPS Implant^®^ Preprosthetic (**c**), and at the 1-year follow-up after primary functional stable dental rehabilitation (**d**)

**Fig. 23:**
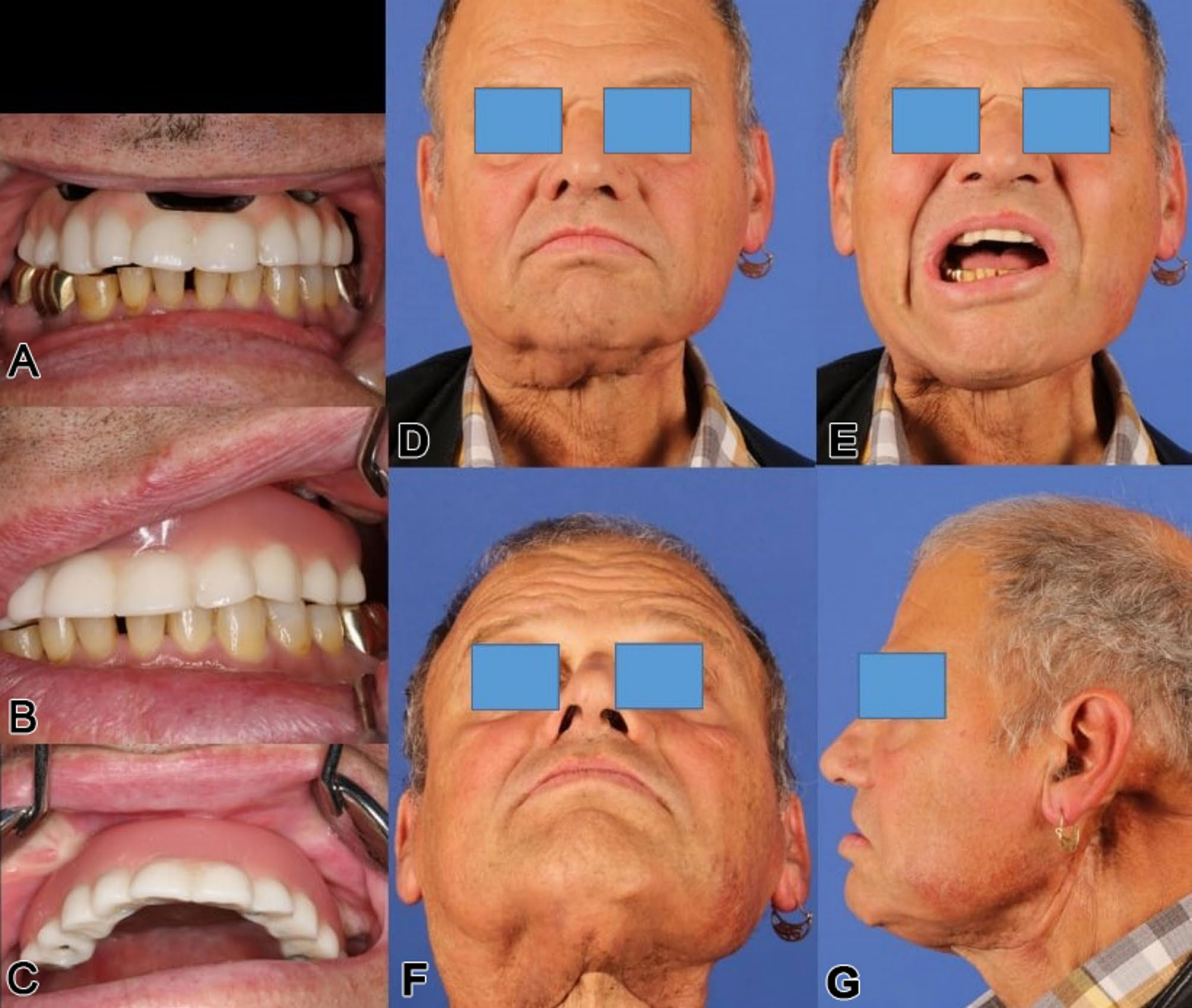
**a – g**: Three intraoral views (on the left) show the first provisional and screwed dental prosthesis in high-water design (top) and the definitive dental prosthesis in occlusion (middle) and with the vestibular shield (bottom) [Annotation: unfortunately, the temporary dental prosthesis was converted into the definitive dental prosthesis, i.e., not designed to be removable, as strongly recommended by the authors]. Four clinical extraoral corresponding views show the patient definitively provided with dental prosthesis after completion of the surgical and adjuvant treatment (including radiotherapy) with evidence of lip competency and, albeit limited, mouth opening; remarkably, there is significant scarring, particularly in the area of cervical metastasis removal on the left side. The severe pseudo-class III malocclusion is compensated for without the need for bone grafting in the resected upper jaw and midface area

At 34 months post-treatment, the patient remains disease-free and fully functional despite his complex condition. Radiographs confirm successful reconstruction with the subperiosteal implant and soft tissue flap, without requiring bone grafting (Figs. 22 and 23). However, the provisional prosthesis was permanently retained rather than replaced with a modular overdenture, complicating hygiene maintenance. Ideally, such structures should be bar-retained or telescope-anchored for better patient care.

## Discussion

The new design considerations for subperiosteal implants may have significant implications for the long-term functionality and stability in patients with severely atrophied or radiotherapy-compromised maxillary structures.

The established buttresses of the upper jaw and midface form a critical foundation for stable osteosynthesis using patient-specific subperiosteal implants, enabling immediate dental rehabilitation [17]. This principle applies to various atrophic conditions, including those seen in cleft lip and palate patients and tooth loss cases [18, 19]. However, for postablative defects, options for achieving stable screw fixation are significantly limited [20]. Compared to standard reconstructions including microvascular bone augmentation combined with conventional dental implants in an at least two stage procedure the presented approach allows for immediate dental rehabilitation. In an anatomically altered situation, with microvascular soft tissue transplants in place the subperiosteal implants do have an advantage over conventional implants in terms of peri-implantitis related complications. The bony buttresses in the cases included in this study, did not allow for zygomatic implants (Figs. 1, 4, 5 and 7). Patients perioperative risk and surgical time can be reduced by avoiding microvascular bone reconstructions. Typically, bone grafts are used to replace missing structures in the upper jaw or midface, enabling implant anchorage for dental rehabilitation [2, 21]. However, the approach presented here avoids bone reconstruction, even in extensive defects, by using design modifications that ensure stable anchorage. This is possible because the upper jaw and midface are solid, non-mobile structures. Rather than inserting bone grafts, the IPS Implants^®^ Preprosthetic’s pillar lengths and directions can be tailored to meet prosthodontic needs, compensating for any pseudo-class III relationship solely through implant design. This innovation offers improved reconstruction, particularly in cases of complete upper jaw or midface loss.

For prosthetic rehabilitation, accurate vertical relation determination is essential [22]. A wax-up and planning dataset using CT or CBCT, with a radiopaque template, may be necessary. Additionally, a defensive contour projection of the upper jaw prosthesis should be carried out based on soft tissue limitations. Patients oral health-related quality of life after dental rehabilitation with IPS Implants^®^ Preprosthetic was evaluated in prior studies and could be proven equally to those with conventional dental implants [23, 24]. The use of additional buttresses for implant fixation is not perceptible by the patient, especially those on the pterygoid process.

To ensure micrometer accuracy, an initial provisional prosthesis was developed in collaboration with a digital lab (Zahntechnisches Labor Duen GmbH, Hamburg, Germany) [16]. A precision-made non-precious metal bar, affixed with acrylic for provisional function, is screw-retained onto the telescoping posts of the maxillary implant (Figs. 11, 19, 20 and 23). This ensures the precise positioning of the posts. The entire workflow, from planning the IPS-Implants^®^ Preprosthetic to the final prosthesis, can be digital, ensuring precision. This quality control element is particularly important in larger frameworks, such as individual footplates, where screw deformation during placement and fixation is a concern.

The footplate design extends onto the intact or partially resected pterygoid process, offering rigid anchorage against the skull base, and positioning closer to the ipsilateral condylar centric point than natural dentition in cases where the dental arch is loaded posteriorly. Real-time navigation can assist in validating the drilling depth, vector, and screw length in the pterygoid area. A paramedian sagittal vector, angling around 45 degrees cranially against the occlusal plane in a supine position, is preferred. FEM analysis in the case displayed in Fig. 1 confirmed the biomechanical advantage of this extension to the pterygoid process for unilateral maxillary implants, offering stronger anchorage than a design without pterygoid support.

The lateral midfacial extension serves as a boom to the lateral skull base, ensuring rigid fixation, even in severe cases of hemi- or bilateral midfacial ablation. In secondary posttraumatic and postablative cases, a running plate maintains midfacial projection after resection, preventing unfavorable soft tissue contraction. This design, inspired by Joseph Gruss’s posttraumatic outer frame reconstruction, extends from the zygomatic arch root, curving over the malar prominence to the subnasal region, even crossing to the contralateral subnasal region. Long running plates, preferably 2.0 plates with 30 or more holes, can be molded on a sterile or patient-specific skull biomodel. Real-time navigation helps define individual drilling depth and vector for stable osteosynthesis at the lateral skull base.

For unilateral anchorage, an intraoral subperiosteal dissection is performed from the malar prominence along the zygomatic arch to the preauricular region, with a limited preauricular incision (approximately 4 cm). A wire loop mounted on the posterior screw hole helps guide the framework safely into position. Self-tapping 2.0 screws (7–9 mm) are used, depending on bone stock, with real-time navigation assisting in screw length and drilling vector determination. The STL design of the implant should be uploaded, ideally automatically, for navigation. This, in combination with the provisional prosthesis as a quality control element, ensures precise surgical execution and quality control.

Key design elements of the IPS Implants^®^ Preprosthetic include:

- A single-piece implant for the upper jaw and midface
- Minimal metal presence near post transitions through soft tissue, based on FEM analyses
- A 3D design to accommodate irregular recipient sites in a suitable, single-fit design
- Extension of the framework far beyond the soft tissue transition, utilizing vascularized strong bones
- Variable thickness of the footplate for biomechanical needs
- Sloped design at the footplate extension’s end
- Multi vector rigid screw fixation

These features, along with cranial base anchorage, make the IPS Implants^®^ Preprosthetic a unique dental rehabilitation device for stable restoration in complex cases, distinguishing it from other subperiosteal implants, particularly those produced by laser melting technology [25–32].

Our study may be limited by its small sample size and its case study based design. However, the general feasibility of the IPS Implants^®^ Preprosthetic is already proven in former work of our group [15, 19, 22]. The high grade of individualization in this treatment option indicates the high variability in defect configuration and diverse patient’s disorders which have to be addressed during preoperative planning and implant design.

Soft tissue considerations remain crucial. The anatomical vertical subunits (cheek, lip, and vestibule) must be fully separated from the horizontal subunit (hard and soft palate) before the insertion of a subperiosteal implant in the upper jaw or midface. This is especially important in scarred or therapy-affected tissues. In Case 3, secondary intervention may be needed to further separate the lip, as observed 23 months post-reconstruction. The non-removable suprastructure may not fully capitalize on the shielding effect of a modular overdenture saddle. In some cases, overcorrecting the vascularized soft tissue envelope, possibly with microvascular free flaps, may be necessary to avoid scarring and tissue shrinking. Adequate soft tissues are essential before IPS-Implants^®^ Preprosthetic insertion to accommodate changes in contour and projection caused by the suprastructure. This is true not only for oncology patients but also for posttraumatic cases (e.g., gunshot wounds, panfacial fractures), where the anatomical requirements for dental rehabilitation may be compromised by overlying soft tissue deficiencies. Posttraumatic tissue impairment can result from repeated surgeries, such as bone grafting or substitute installations, which lead to scarring in atrophic patients. In the case of ectodermal dysplasia, no additional soft tissue transplantation was needed, and despite a history of multiple failed interventions, the IPS Implants^®^ Preprosthetic withstood the biomechanical challenge of a severe pseudo-class III condition exacerbated by the excessive use of conventional dental implants in the lower jaw.

## Conclusion

The advanced IPS Implants^®^ Preprosthetic design introduces new possibilities for stable anchorage to the cranial vault or skull base, allowing for biomechanical force absorption in cases of extensive bilateral midfacial or upper jaw defects. Soft tissue reconstructions, combined with the IPS Implants^®^ Preprosthetic and overdenture, are sufficient for rehabilitation in severe bone loss cases, regardless of pseudo-class III severity. This approach offers a new treatment modality for reliable dental rehabilitation in challenging cases, without the need for extensive bone grafting procedures.

## Data Availability

The datasets generated and analyzed during the current study are not publicly available due to institutional restrictions but are available from the corresponding author upon reasonable request.
